# Efficacy and safety of adjunctive padsevonil in adults with drug‐resistant focal epilepsy: Results from two double‐blind, randomized, placebo‐controlled trials

**DOI:** 10.1002/epi4.12656

**Published:** 2022-10-22

**Authors:** Michael Rademacher, Manuel Toledo, Wim Van Paesschen, Kore K. Liow, Ivan G. Milanov, Maria‐Luise Esch, Nan Wang, Merran MacPherson, William J. Byrnes, Timothy D. C. Minh, Elizabeth Webster, Konrad J. Werhahn

**Affiliations:** ^1^ Department of Epileptology University of Bonn Medical Center Bonn Germany; ^2^ Epilepsy Unit, Neurology Department Vall d'Hebron University Hospital Barcelona Spain; ^3^ Department of Neurology University Hospitals Leuven Leuven Belgium; ^4^ Comprehensive Epilepsy Center Hawaii Pacific Neuroscience Honolulu Hawaii USA; ^5^ Medical University of Sofia Sofia Bulgaria; ^6^ UCB Pharma Monheim am Rhein Germany; ^7^ UCB Pharma Morrisville North Carolina USA; ^8^ UCB Pharma Slough UK

**Keywords:** antiepileptic drug, antiseizure medication, dual mechanism of action, focal seizure, synaptic vesicle protein 2, tolerability

## Abstract

**Objective:**

To characterize efficacy, safety/tolerability, and pharmacokinetics of padsevonil (PSL) administered concomitantly with ≤3 antiseizure medications (ASMs) for observable focal seizures in adults with drug‐resistant epilepsy in two multicenter, randomized, double‐blind, placebo‐controlled, parallel‐group trials.

**Methods:**

The phase 2b dose‐finding trial (EP0091/NCT03373383) randomized patients 1:1:1:1:1 to PSL 50/100/200/400 mg or placebo twice daily (b.i.d.). The phase 3 efficacy trial (EP0092/NCT03739840) randomized patients 1:1:1:1 to PSL 100/200/400 mg or placebo b.i.d. Patients with observable (focal aware with motor symptoms, focal impaired awareness, focal to bilateral tonic–clonic) focal seizures for ≥3 years, experiencing them ≥4 times per 28 days including during the 4‐week baseline period despite treatment with ≥4 lifetime ASMs including current ASMs, were enrolled.

**Results:**

In EP0091 and EP0092, 410 and 231 patients, respectively, were randomized and received at least one dose of trial medication. In patients in EP0091 on PSL 50/100/200/400 mg b.i.d. (*n* = 80/82/81/81, respectively) versus placebo (*n* = 81), outcomes included percentage reductions over placebo in observable focal seizure frequency during the 12‐week maintenance period: 17.2%, 19.1% (*p* = 0.128), 19.2% (*p* = 0.128), 12.4% (*p* = 0.248); 75% responder rates (*p*‐values for odds ratios): 13.8%, 12.2% (*p* = 0.192), 11.1% (*p* = 0.192), 16.0% (*p* = 0.124) versus 6.2%; 50% responder rates: 33.8% (*p* = 0.045), 31.7% (*p* = 0.079), 25.9% (*p* = 0.338), 32.1% (*p* = 0.087), versus 21.0%; TEAEs were reported by 82.7% (67/81), 78.3% (65/83), 74.4% (61/82), 90.1% (73/81) versus 78.3% (65/83). In patients in EP0092 on PSL 100/200/400 mg b.i.d. (*n* = 60/56/56, respectively) versus placebo (*n* = 54), outcomes included percentage reductions over placebo: −5.6% (*p* = 0.687), 6.5% (*p* = 0.687), 6.3% (*p* = 0.687); 75% responder rates: 15.3% (*p* = 0.989), 12.5% (*p* = 0.989), 14.3% (*p* = 0.989) versus 13.0%; 50% responder rates: 35.6% (*p* = 0.425), 33.9% (*p* = 0.625), and 42.9% (*p* = 0.125) versus 27.8%; TEAEs were reported by 80.0% (48/60), 78.9% (45/57), 83.1% (49/59) versus 67.3% (37/55).

**Significance:**

In both trials, the primary outcomes did not reach statistical significance in any PSL dose group compared with placebo. PSL was generally well tolerated, and no new safety signals were identified.

**Key Points**

- Two multicenter, randomized, double‐blind, placebo‐controlled, parallel‐group trials of adjunctive padsevonil for observable focal seizures.
- Patients represented a severely affected population with drug‐resistant epilepsy (median of 11 observable focal seizures per 28 days).
- In both trials, the primary outcomes did not reach statistical significance in any padsevonil dose group compared with placebo.
- Numerical improvements were observed for the padsevonil dose groups compared with placebo.
- Padsevonil was generally well tolerated, and no new safety signals were identified.

## 1 INTRODUCTION

During the past few decades, antiseizure medications (ASMs) with a range of different mechanisms of action have become available, some with an improved pharmacokinetic (PK) and tolerability profile over others. However, no ASM has demonstrated superior efficacy over other ASMs in adequate well‐controlled trials. Consequently, the proportion of patients with active epilepsy and multiple ASM resistance has not changed.^1^, ^2^ The likelihood of achieving seizure freedom is reduced with each unsuccessful ASM regimen.^3^, ^4^ Drug‐resistant epilepsy (affecting 30%–40% of patients) is associated with significant morbidity and increased risk of mortality.^5^, ^6^, ^7^, ^8^ There continues to be a need for novel agents providing improvements in ASM effectiveness.^5^, ^8^

Padsevonil (PSL) is a first‐in‐class ASM candidate designed uniquely with both pre‐ and postsynaptic therapeutic mechanisms. Unlike many available ASMs, PSL was developed in a target‐based, rational drug design program that combined presynaptic interaction with synaptic vesicle protein 2 [SV2] isoforms (equally high affinity to SV2A, SV2B, and SV2C) with the postsynaptic enhancement of GABAergic inhibition (moderate affinity at the benzodiazepine site of the γ‐aminobutyric acid type A [GABA_A_] receptor).^9^, ^10^, ^11^ PSL binds SV2A with an affinity that is approximately 2000‐ and 100‐fold greater than that of levetiracetam (LEV) and brivaracetam (BRV), respectively.^10^, ^11^ Like many benzodiazepines, PSL binds the GABA_A_ receptor between the α1 and γ2 subunits.^11^, ^12^ This is distinct from clobazam, which binds between the α2 and γ2 subunits of the GABA_A_ receptor, decreasing the likelihood of sedation.^13^, ^14^ PSL was specifically designed for increased anticonvulsant activity, with the rationale to test the drug in a population with the highest unmet medical need (patients with drug‐resistant seizures). This novel pre‐ and postsynaptic mechanism of PSL conferred robust efficacy in multiple nonclinical models.^15^

In a phase 2a proof‐of‐concept trial in adults with drug‐resistant focal seizures (EP0069; NCT02495844), PSL was associated with a favorable safety profile and clinically meaningful reductions in seizure frequency compared with placebo.^9^ When the data from the proof‐of‐concept trial seem to corroborate the impression of high anticonvulsive efficacy in patients with focal epilepsy, the sponsor started two large trials (phase 2b and phase 3 trials) in parallel. The purpose of this article is to report data from these two trials and to discuss the impact of the results on the clinical development of PSL.

In the dose‐finding phase 2b trial, the primary objectives were to characterize the dose–response relationship with respect to the efficacy of PSL administered concomitantly with up to three ASMs for observable focal seizures in patients with drug‐resistant epilepsy and to evaluate the efficacy of PSL (50, 100, 200, 400 mg twice daily [b.i.d.]) compared with placebo. The secondary objective was to assess the safety and tolerability of all doses of PSL in relation to placebo. The PK objectives were to evaluate the steady‐state PK profiles of PSL and the desmethyl metabolite, the effect of enzyme‐inducing concomitant ASMs on PSL exposure, and the concomitant ASM (and/or relevant metabolites) plasma levels. The desmethyl metabolite is a major circulating active metabolite of PSL that has at least 10‐fold lower affinity across SV2 subtypes compared with PSL itself and has low affinity for central benzodiazepine receptor.

In the phase 3 efficacy trial, the primary objective was to evaluate the efficacy of PSL (100, 200, 400 mg b.i.d.) administered concomitantly with up to three ASMs compared with placebo for observable focal seizures in patients with drug‐resistant epilepsy. The secondary objective was to assess the safety and tolerability of PSL in relation to placebo.

## 2 METHODS

### 2.1 Overall trial designs and patients

The dose‐finding trial (EP0091; NCT03373383; ARISE) was a phase 2b, multicenter, randomized, double‐blind, placebo‐controlled, parallel‐group trial in which eligible patients were randomized 1:1:1:1:1 to PSL 50, 100, 200, 400 mg, or placebo b.i.d. (Figure S1). The phase 3 efficacy trial (EP0092; NCT03739840; DUET) was a multicenter, randomized, double‐blind, placebo‐controlled, parallel‐group trial in which eligible patients were randomized 1:1:1:1 to PSL 100, 200, 400 mg, or placebo b.i.d. (Figure S2). Both trials were quadruple masked (patient, care provider, Investigator, outcomes assessor), and conducted in North America, Europe, and the Asia‐Pacific regions in adults (≥18 years of age weighing ≥40 kg) with drug‐resistant epilepsy and uncontrolled focal seizures. The patients were outpatients recruited from secondary and tertiary care centers.

The trial design and patient eligibility were the same for both trials, except for the randomized target dose levels of PSL. Patients were eligible to enroll in either trial if they had a diagnosis of epilepsy and had observable (focal aware with motor symptoms, focal impaired awareness, and focal to bilateral tonic–clonic) focal seizures for ≥3 years at the time of enrollment as documented by electroencephalogram. A brain magnetic resonance imaging examination was to be performed before randomization if not carried out in the past 10 years. Eligible patients had experienced at least four spontaneous and observable focal seizures per 28 days, with at least one during each 4‐week interval of the 8 weeks before the screening visit, and at least four during the baseline period, despite treatment with at least four lifetime ASMs including current ASMs. Exclusion criteria are reported in Appendix S1.

Patients were receiving a stable regimen of one to three concomitant ASMs (with or without neurostimulation devices including vagus nerve stimulators) during the 8 weeks before the screening visit and throughout the trial. LEV, BRV, and clobazam were among the concomitant ASMs that were permitted (see Appendix S1 for exclusions). Settings for neurostimulation devices should have remained stable for 12 weeks before the screening visit and throughout the trial.

During the screening visit, patients (or their legal representative) provided their written informed consent, after which they entered a 4‐week baseline period. Patients were randomized (random permuted blocks using interactive response technology to assign patients to interventions) stratified by current use of ASMs with binding to SV2A proteins (LEV or BRV) and by region (EP0091: North America, Europe and Australia, Asia; EP0092: North America, Europe and Australia, Japan). PSL was initiated at 50 mg b.i.d. and up‐titrated over 3 weeks in the titration period, which was followed by a 1‐week stabilization period. During the stabilization period and ≥2 days before the start of the maintenance period, one dose reduction was allowed for tolerability reasons (to 25, 75, 150, 300 mg b.i.d. in patients randomized to 50, 100, 200, 400 mg b.i.d. PSL, respectively; Figures S1 and S2). Patients were withdrawn if they could not tolerate the trial medication during the titration period, or if they experienced tolerability issues at the target dose during stabilization and could not tolerate the reduced dose.

Patients then entered the 12‐week maintenance period during which no further dose adjustments were permitted. Those who completed the maintenance period could enroll in an open‐label extension trial (EP0093; NCT03370120) and entered a 3‐week blinded conversion period where doses were adapted to reach 200 mg b.i.d. PSL. Patients who withdrew or who did not enter the open‐label extension trial entered a 3‐week taper period (which may have been faster or slower if medically necessary), followed by a 1‐week drug‐free period. Patients not entering the open‐label extension trial had an echocardiogram at the safety follow‐up visit 30 days after the last dose of trial medication, and 6 months (±1 month) after the last dose of trial medication in patients who received the trial medication for >3 weeks. Echocardiograms were conducted as a precaution based on the presence of some minor asymptomatic, focal cardiac valvular, and epicardial inflammatory lesions in dogs in a 39‐week toxicity study; these lesions were deemed most likely not relevant for humans by an independent assessment (data on file).

The trials were conducted in accordance with the International Council on Harmonization guidelines on Good Clinical Practice, the Declaration of Helsinki, and local laws. The trial protocols, amendments, and patient‐informed consent forms were reviewed by national, regional, or independent ethics committees or institutional review boards.

### 2.2 Outcomes in both trials

Primary efficacy outcomes were the change in log‐transformed observable focal seizure frequency from baseline and 75% responder rate (≥75% reduction in observable focal seizure frequency from baseline) over the 12‐week maintenance period. Seizure frequency was adjusted to a 28‐day frequency. Secondary efficacy outcomes were 50% responder rate (≥50% reduction in observable focal seizure frequency from baseline) and percentage reduction in observable focal seizure frequency per 28 days from baseline over the 12‐week maintenance period. Other efficacy outcomes included seizure freedom during the 12‐week maintenance period, defined as patients who did not report any seizures of any type (focal, generalized, and unclassified epileptic seizures), completed the maintenance period, and did not have any days of missing diary entries over the maintenance period.

Primary safety outcomes were the incidence of treatment‐emergent adverse events (TEAEs), TEAEs leading to discontinuation, and serious TEAEs. Other safety outcomes included physical and neurological examinations, psychiatric monitoring, and diagnostic tests (e.g., 12‐lead electrocardiograms and echocardiograms).

In the dose‐finding trial, PK outcomes included blood concentrations of PSL, the desmethyl metabolite, and plasma concentrations of concomitantly administered ASMs.

### 2.3 Statistical analyses

Four‐hundred patients were planned to be randomized in the dose‐finding trial, and 500 were planned to be randomized in the phase 3 efficacy trial (400 in the Europe/North America/Japan regions, and 100 in China). In both trials, safety outcomes were assessed in the Safety Set (SS), which included all patients who received at least one dose or the partial dose of trial medication, and efficacy outcomes were assessed in the Full Analysis Set (FAS), which included all SS patients who had baseline and postbaseline seizure frequency data during the treatment period.

In the dose‐finding trial, PK outcomes were assessed in the Pharmacokinetic Per‐Protocol Set (PK‐PPS) or the ASM‐PK‐PPS, which included patients with at least one PSL trough blood concentration measurement or ASM trough plasma concentration measurement, respectively, who did not have deviations affecting the assessment of steady‐state concentrations. Steady‐state blood concentrations of PSL and the desmethyl metabolite at the 12‐h time point (just before the next dose) were summarized at weeks 2, 4, 8, 12, and 16 following PSL treatment commencement. Plasma concentration data for concomitantly administered ASMs, collected before PSL treatment and at weeks 12 and 16 following the start of PSL treatment, were assessed by evaluating ratios of steady‐state levels during the 12‐week maintenance period versus baseline levels, using mixed models.

Statistical analysis was performed using SAS® Version 9.3. An analysis of covariance (ANCOVA) model was applied for change in seizure frequency, and a logistic regression was applied for the analysis of the responder rates. Unless otherwise specified, statistical tests were two‐sided and were performed at the 0.05 level of significance. For full details see Appendix S2. Additional prespecified analyses were conducted by use of ASMs with binding to SV2A proteins (LEV and/or BRV) at trial entry.

## 3 RESULTS

### 3.1 The dose‐finding trial

#### 3.1.1 Baseline demographics and disposition

The dose‐finding trial was conducted between February 2018 and January 2020, at 148 sites in 19 countries. 411 patients were randomized to receive placebo (83 patients), PSL 50 mg b.i.d. (81 patients), PSL 100 mg b.i.d. (83 patients), PSL 200 mg b.i.d. (82 patients), or PSL 400 mg b.i.d. (82 patients). One patient in the PSL 400 mg b.i.d. group withdrew consent before receiving trial medication. Therefore, 410 patients were treated (SS), of whom 322 (78.5%) completed the trial (83.1% for placebo and 77.4% for all PSL patients) and 88 (21.5%) discontinued (16.9% for placebo and 22.6% for all PSL patients; Figure S3). The most common primary reason for discontinuation was adverse events (60 [14.6%]; 8.4% for placebo and 16.2% for all PSL patients). Patients represented a severely affected population with high baseline seizure frequency (median of 11.0 observable focal seizures per 28 days) and high number of lifetime ASMs (52.1% of patients with ≥8 lifetime ASMs) (Table 1).

**TABLE 1:** Trial EP0091 baseline demographics, epilepsy characteristics, lifetime and concomitant ASMs (full analysis set)

	Placebo (*n* = 81)	PSL 50 mg b.i.d. (*n* = 80)	PSL 100 mg b.i.d. (*n* = 82)	PSL 200 mg b.i.d. (*n* = 81)	PSL 400 mg b.i.d. (*n* = 81)	All patients (*n* = 405)
Patient demographics						
Age, mean (SD), years	39.8 (13.0)	42.3 (11.5)	36.7 (13.0)	40.9 (12.0)	38.9 (12.1)	39.7 (12.4)
Female, *n* (%)	48 (59.3)	46 (57.5)	47 (57.3)	50 (61.7)	43 (53.1)	234 (57.8)
Epilepsy characteristics						
Duration of epilepsy, mean (SD), years	22.2 (13.1)	25.8 (13.0)	22.3 (12.4)	24.7 (13.6)	24.3 (12.0)	23.9 (12.8)
Age at onset of epilepsy, mean (SD), years	18.3 (13.1)	17.4 (12.4)	15.0 (11.5)	17.0 (11.8)	15.3 (11.8)	16.6 (12.1)
VNS at screening, *n* (%)	12 (14.8)	15 (18.8)	17 (20.7)	15 (18.5)	22 (27.2)	81 (20.0)
Evaluated for epilepsy surgery, *n* (%)	35 (43.2)	47 (58.8)	46 (56.1)	28 (34.6)	51 (63.0)	207 (51.1)
Baseline focal seizure^a^ frequency per 28 days, median (range)	10.5 (3.3, 256.0)	12.1 (3.7, 250.1)	10.6 (1.0, 886.0)	11.6 (3.2, 276.7)	9.0 (3.2, 810.0)	11.0 (1.0, 886.0)
Seizure classification history at any time before trial entry, *n* (%)						
Any focal seizures	81 (100)	79 (98.8)	82 (100)	81 (100)	81 (100)	404 (99.8)
Focal aware	27 (33.3)	41 (51.3)	39 (47.6)	32 (39.5)	42 (51.9)	181 (44.7)
With motor symptoms	13 (16.0)	24 (30.0)	22 (26.8)	17 (21.0)	19 (23.5)	95 (23.5)
Focal impaired awareness	67 (82.7)	73 (91.3)	74 (90.2)	75 (92.6)	67 (82.7)	356 (87.9)
Focal to bilateral tonic–clonic	50 (61.7)	49 (61.3)	47 (57.3)	51 (63.0)	49 (60.5)	246 (60.7)
Any generalized seizures	2 (2.5)	5 (6.3)	3 (3.7)	2 (2.5)	3 (3.7)	15 (3.7)
Number of lifetime ASMs, *n* (%)^b^						
<4	0	1 (1.3)	0	2 (2.5)	0	3 (0.7)
4–5	20 (24.7)	15 (18.8)	24 (29.3)	20 (24.7)	19 (23.5)	98 (24.2)
6–7	16 (19.8)	22 (27.5)	18 (22.0)	19 (23.5)	18 (22.2)	93 (23.0)
8–10	27 (33.3)	18 (22.5)	19 (23.2)	19 (23.5)	25 (30.9)	108 (26.7)
>10	18 (22.2)	24 (30.0)	21 (25.6)	21 (25.9)	19 (23.5)	103 (25.4)
Number of ASMs taken at trial entry, *n* (%)						
1	9 (11.1)	7 (8.8)	7 (8.5)	4 (4.9)	7 (8.6)	34 (8.4)
2	36 (44.4)	32 (40.0)	33 (40.2)	36 (44.4)	36 (44.4)	173 (42.7)
3	34 (42.0)	34 (42.5)	41 (50.0)	36 (44.4)	36 (44.4)	181 (44.7)
>3	2 (2.5)	7 (8.8)	1 (1.2)	5 (6.2)	2 (2.5)	17 (4.2)
Concomitant ASMs taken by ≥15% of all patients, *n* (%)						
Lacosamide	33 (40.7)	32 (40.0)	39 (47.6)	32 (39.5)	36 (44.4)	172 (42.5)
Levetiracetam	27 (33.3)	28 (35.0)	34 (41.5)	30 (37.0)	35 (43.2)	154 (38.0)
Lamotrigine	26 (32.1)	24 (30.0)	26 (31.7)	29 (35.8)	33 (40.7)	138 (34.1)
Valproate^c^	19 (23.5)	26 (32.5)	20 (24.4)	23 (28.4)	12 (14.8)	100 (24.7)
Oxcarbazepine	12 (14.8)	19 (23.8)	20 (24.4)	14 (17.3)	10 (12.3)	75 (18.5)
Perampanel	16 (19.8)	15 (18.8)	15 (18.3)	12 (14.8)	11 (13.6)	69 (17.0)
Brivaracetam	12 (14.8)	14 (17.5)	10 (12.2)	19 (23.5)	10 (12.3)	65 (16.0)

Abbreviations: ASM, antiseizure medication; b.i.d., twice daily; PSL, padsevonil; SD, standard deviation; VNS, vagus nerve stimulation.

^a^ Includes the following seizure types: focal aware with motor symptoms, focal impaired awareness, focal to bilateral tonic–clonic.

^b^ Includes previous ASMs and ASMs taken at trial entry.

^c^ Includes ergenyl chrono, valproate magnesium, valproate semisodium, valproate sodium, and valproic acid.

#### 3.1.2 Efficacy

**FIGURE 1:**
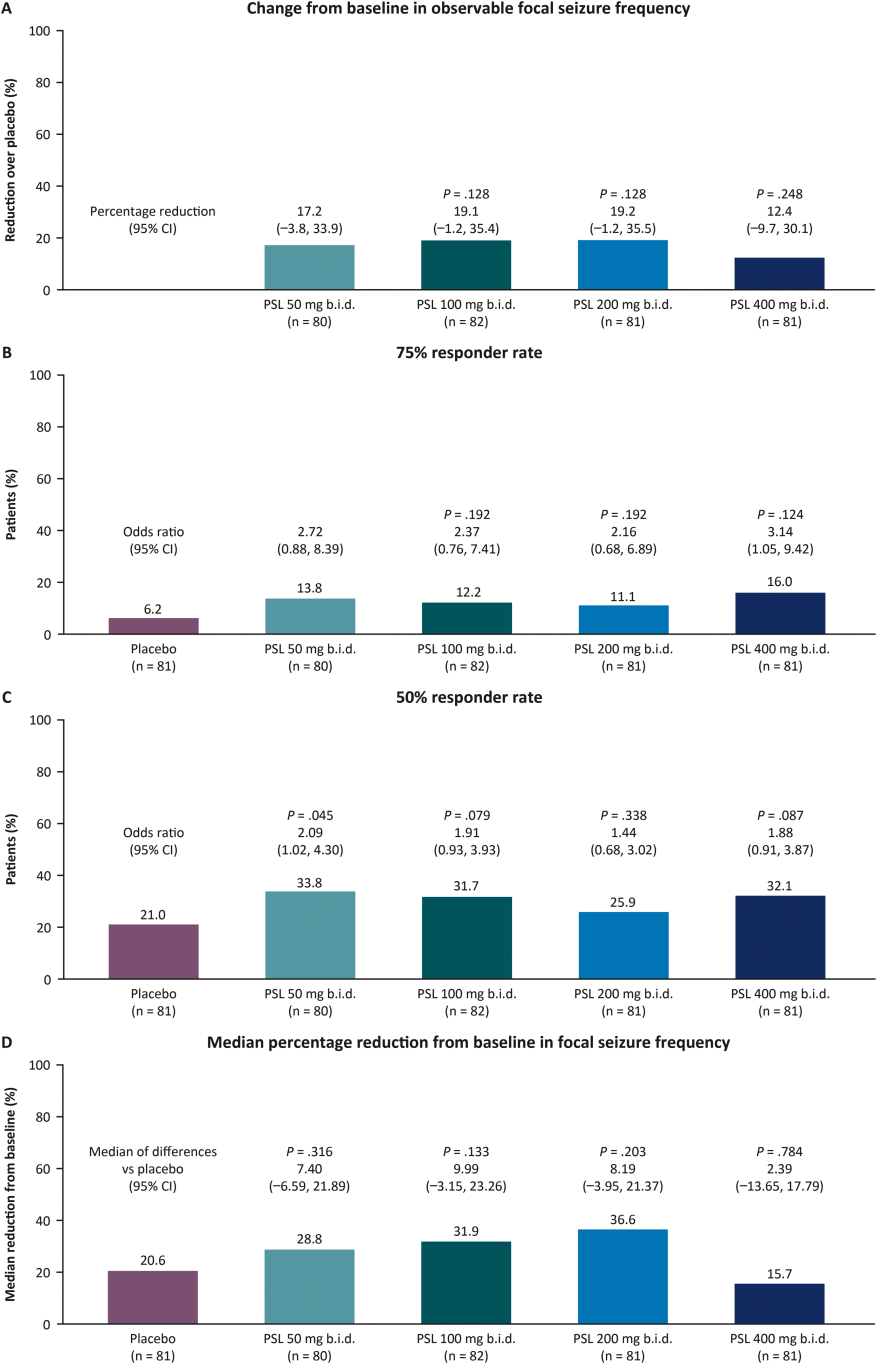
Efficacy outcomes of patients randomized to placebo or PSL by randomized dose group for observable focal seizures over the 12‐week maintenance period in the dose‐finding trial (EP0091; full analysis set): (A) change from baseline in log‐transformed observable focal seizure frequency as percentage reductions over placebo; (B) 75% responder rate; (C) 50% responder rate; (D) median percentage reduction from baseline in focal seizures per 28 days. For the primary outcomes (percentage reductions over placebo in observable focal seizure frequency and 75% responder rates), statistical comparison of the PSL 50 mg b.i.d. dose group to placebo was provided only if the other three higher doses were significant. ASM, antiseizure medication; b.i.d., twice daily; PSL, padsevonil.

Four‐hundred and five patients had baseline and postbaseline seizure frequency data and were included in efficacy assessments. The least squares mean change in log‐transformed observable focal seizure frequency from baseline over the 12‐week maintenance period was −0.46, −0.49, −0.49, −0.41 with PSL 50, 100, 200, 400 mg b.i.d. versus −0.28 with placebo. In patients on PSL 50, 100, 200, and 400 mg b.i.d., percentage reductions over placebo (and adjusted *p*‐values) from baseline during the 12‐week maintenance period were 17.2%, 19.1% (*p* = 0.128), 19.2% (*p* = 0.128), and 12.4% (*p* = 0.248), respectively (Figure 1A), and 75% responder rates (and adjusted *p*‐values for odds ratios of PSL versus placebo) were 13.8%, 12.2% (*p* = 0.192), 11.1% (*p* = 0.192), and 16.0% (*p* = 0.124), versus placebo (6.2%) (Figure 1B). 50% responder rates ranged from 25.9% to 33.8% in PSL dose groups, and were different for the PSL 50 mg b.i.d. group (33.8%) versus placebo (21.0%; nominal *p*‐value for odds ratio of PSL versus placebo not adjusted for multiplicity: *p* = 0.045) (Figure 1C). Median percentage reductions from baseline in observable focal seizure frequency per 28 days ranged from 15.7% to 36.6% with PSL versus 20.6% with placebo (Figure 1D). Among patients who completed the maintenance period, 6.1%, 1.5%, 8.2%, and 6.9% of patients on PSL 50 (*n* = 66), 100 (*n* = 68), 200 (*n* = 61), and 400 mg b.i.d. (*n* = 58), respectively, versus 0% on placebo (*n* = 70) were free from all seizures.

#### 3.1.3 Subgroup analyses

Randomization was stratified by current use (yes/no) of ASMs with binding to SV2A proteins (LEV and/or BRV). Because “yes” was indicated by 188/411 patients (45.7%, Randomized Set) while a total of 217 patients were recorded to have taken LEV (*n* = 154) or BRV (*n* = 63) as ASMs at trial entry (FAS), prespecified sensitivity analyses were performed to account for this difference.

In patients who were recorded to have been taking LEV or BRV at trial entry, nominally small improvements over placebo were generally observed across PSL doses (Appendix S3). In patients who were recorded as not taking LEV or BRV at trial entry, there were generally consistent numerical improvements over placebo in log‐transformed observable focal seizure frequency from baseline, and nominally higher 75% responder rates in patients taking PSL compared with placebo (Appendix S3).

#### 3.1.4 Tolerability

The overall incidences of TEAEs were generally similar across the PSL and placebo treatment groups (Table 2; SS). There was no dose‐dependency in the incidence of TEAEs up to PSL 200 mg b.i.d. (range 74.4%–82.7%), and the highest incidence was observed in the PSL 400 mg b.i.d. group (90.1%). Discontinuations due to TEAEs were more common with PSL (15.9% overall) than placebo (8.4%), and incidence generally increased with increasing dose. The most common TEAEs with PSL (≥10% of all PSL patients) were somnolence (30.0%), dizziness (26.9%), fatigue (20.2%), and headache (15.3%) (placebo: 12.0%, 10.8%, 12.0%, and 12.0%, respectively), with incidences generally higher in PSL dose groups compared with the placebo group.

**TABLE 2:** Trial EP0091 incidence of overall TEAEs and most common TEAEs with onset during the treatment period (safety set)

	Placebo (*n* = 83)	PSL 50 mg b.i.d. (*n* = 81)	PSL 100 mg b.i.d. (*n* = 83)	PSL 200 mg b.i.d. (*n* = 82)	PSL 400 mg b.i.d. (*n* = 81)	All PSL patients (*n* = 327)
Any TEAEs, *n* (%)	65 (78.3)	67 (82.7)	65 (78.3)	61 (74.4)	73 (90.1)	266 (81.3)
Drug‐related TEAEs	36 (43.4)	47 (58.0)	49 (59.0)	50 (61.0)	65 (80.2)	211 (64.5)
Serious TEAEs	3 (3.6)	5 (6.2)	4 (4.8)	3 (3.7)	5 (6.2)	17 (5.2)
Severe TEAEs	7 (8.4)	6 (7.4)	6 (7.2)	7 (8.5)	7 (8.6)	26 (8.0)
Discontinuations due to TEAEs	7 (8.4)	6 (7.4)	10 (12.0)	15 (18.3)	21 (25.9)	52 (15.9)
Deaths	0	0	0	0	0	0
TEAEs^a^ reported by ≥5% of all patients on placebo or PSL, *n* (%)						
Somnolence	10 (12.0)	19 (23.5)	24 (28.9)	25 (30.5)	30 (37.0)	98 (30.0)
Dizziness	9 (10.8)	18 (22.2)	23 (27.7)	19 (23.2)	28 (34.6)	88 (26.9)
Fatigue	10 (12.0)	20 (24.7)	12 (14.5)	14 (17.1)	20 (24.7)	66 (20.2)
Headache	10 (12.0)	17 (21.0)	9 (10.8)	15 (18.3)	9 (11.1)	50 (15.3)
Nasopharyngitis	5 (6.0)	5 (6.2)	9 (10.8)	7 (8.5)	5 (6.2)	26 (8.0)
Memory impairment	1 (1.2)	3 (3.7)	1 (1.2)	8 (9.8)	14 (17.3)	26 (8.0)
Nausea	7 (8.4)	4 (4.9)	7 (8.4)	2 (2.4)	7 (8.6)	20 (6.1)
Diarrhea	3 (3.6)	6 (7.4)	3 (3.6)	5 (6.1)	4 (4.9)	18 (5.5)
Tremor	0	5 (6.2)	2 (2.4)	6 (7.3)	5 (6.2)	18 (5.5)
Irritability	8 (9.6)	4 (4.9)	7 (8.4)	1 (1.2)	5 (6.2)	17 (5.2)
Vertigo	7 (8.4)	3 (3.7)	2 (2.4)	3 (3.7)	4 (4.9)	12 (3.7)

Abbreviations: b.i.d., twice daily; PSL, padsevonil; TEAE, treatment‐emergent adverse event.

^a^ Medical Dictionary for Regulatory Activities Version 22.1 Preferred Term.

The incidences of serious TEAEs were generally low and similar across treatment groups (range 3.6%–6.2%; 3.6% for placebo). The majority of serious TEAEs were considered not related to PSL. During the treatment period, three (0.9%) patients across all PSL groups reported four serious TEAEs that were considered drug‐related; one patient had a head injury during titration and stabilization (drug interrupted, resolving at the time of reporting), another had altered state of consciousness and syncope during titration (drug withdrawn, resolved), and one patient had status epilepticus during maintenance (drug withdrawn, resolved). Most TEAEs were mild or moderate in intensity, and few patients reported severe TEAEs. No clinically relevant differences between treatment groups were observed for any changes from baseline in hematology, blood chemistry, urinary parameters, vital signs, electrocardiograms, and echocardiograms. There were no new safety signals identified.

#### 3.1.5 Clinical pharmacology

Steady‐state trough concentrations of PSL and the desmethyl metabolite were maintained over the 16‐week treatment period and were as anticipated for the doses of PSL administered. Although there was overlap in exposure among the PSL doses, PSL exposure increased with increasing dose, and differentiation in exposure was observed between the PSL 50 mg b.i.d. and PSL 400 mg b.i.d. groups (Figure S4). Treatment with PSL had no clinically relevant effect on the ASM plasma trough concentrations of BRV, LEV, eslicarbazepine, oxcarbazepine, lacosamide, valproate, and zonisamide (Figure S5).

### 3.2 The phase 3 efficacy trial

#### 3.2.1 Disposition, baseline demographics, and epilepsy characteristics

The phase 3 efficacy trial was conducted between March 2019 and September 2020, at 141 sites in 28 countries. The phase 3 efficacy trial temporarily paused enrollment of new trial patients because of the coronavirus disease 2019 (COVID‐19) pandemic before it was terminated on September 28, 2020 (date of last patient last visit). 232 patients were enrolled and randomized to receive placebo (56 patients), PSL 100 mg b.i.d. (60 patients), PSL 200 mg b.i.d. (57 patients), or PSL 400 mg b.i.d. (59 patients) (Figure S6). Of these, one patient in the placebo group did not receive any trial medication (discontinued due to adverse event during the baseline period) and was excluded from the SS. These patients represented a severely affected population with a high baseline seizure frequency (median of 11.0 observable focal seizures per 28 days) (Table 3).

**TABLE 3:** Trial EP0092 baseline demographics, epilepsy characteristics, lifetime and concomitant ASMs (full analysis set)

	Placebo (*n* = 54)	PSL 100 mg b.i.d. (*n* = 60)	PSL 200 mg b.i.d. (*n* = 56)	PSL 400 mg b.i.d. (*n* = 56)	All patients (*n* = 226)
Patient demographics					
Age, mean (SD), years	41.3 (13.3)	40.7 (13.0)	39.8 (14.3)	39.8 (13.7)	40.4 (13.5)
Female, *n* (%)	32 (59.3)	34 (56.7)	28 (50.0)	32 (57.1)	126 (55.8)
Epilepsy characteristics					
Duration of epilepsy, mean (SD), years	26.6 (13.7)	21.8 (13.1)	26.4 (13.8)	23.1 (12.5)	24.4 (13.3)
Age at onset of epilepsy, mean (SD), years	15.5 (12.9)	19.6 (13.3)	14.1 (10.6)	17.5 (14.4)	16.7 (13.0)
VNS at screening, *n* (%)	10 (18.5)	9 (15.0)	6 (10.7)	13 (23.2)	38 (16.8)
Evaluated for epilepsy surgery, *n* (%)	26 (48.1)	22 (36.7)	18 (32.1)	29 (51.8)	95 (42.0)
Baseline focal seizure^a^ frequency per 28 days, median (range)	15.8 (3.6, 390.1)	11.0 (3.7, 335.1)	10.6 (3.0, 133.7)	10.0 (3.6, 231.7)	11.0 (3.0, 390.1)
Seizure classification history at any time before trial entry, *n* (%)					
Any focal seizures	54 (100)	60 (100)	56 (100)	56 (100)	226 (100)
Focal aware	21 (38.9)	33 (55.0)	27 (48.2)	31 (55.4)	112 (49.6)
With motor symptoms	13 (24.1)	21 (35.0)	15 (26.8)	19 (33.9)	68 (30.1)
Focal impaired awareness	46 (85.2)	47 (78.3)	48 (85.7)	50 (89.3)	191 (84.5)
Focal to bilateral tonic–clonic	35 (64.8)	38 (63.3)	35 (62.5)	34 (60.7)	142 (62.8)
Any generalized seizures	0	0	0	0	0
Number of lifetime ASMs, median (range)^b^					
<4	2 (3.7)	2 (3.3)	4 (7.1)	1 (1.8)	9 (4.0)
4–5	14 (25.9)	19 (31.7)	22 (39.3)	9 (16.1)	64 (28.3)
6–7	13 (24.1)	18 (30.0)	15 (26.8)	20 (35.7)	66 (29.2)
8–10	16 (29.6)	13 (21.7)	9 (16.1)	13 (23.2)	51 (22.6)
>10	9 (16.7)	8 (13.3)	6 (10.7)	13 (23.2)	36 (15.9)
Number of ASMs taken at trial entry, *n* (%)					
1	2 (3.7)	7 (11.7)	7 (12.5)	5 (8.9)	21 (9.3)
2	23 (42.6)	21 (35.0)	17 (30.4)	28 (50.0)	89 (39.4)
3	25 (46.3)	29 (48.3)	29 (51.8)	23 (41.1)	106 (46.9)
>3	4 (7.4)	3 (5.0)	3 (5.4)	0	10 (4.4)
Concomitant ASMs taken by ≥15% of all patients, *n* (%)					
Levetiracetam	24 (44.4)	21 (35.0)	24 (42.9)	23 (41.1)	92 (40.7)
Lacosamide	20 (37.0)	24 (40.0)	16 (28.6)	19 (33.9)	79 (35.0)
Lamotrigine	18 (33.3)	16 (26.7)	21 (37.5)	18 (32.1)	73 (32.3)
Valproate sodium	12 (22.2)	17 (28.3)	15 (26.8)	11 (19.6)	55 (24.3)
Oxcarbazepine	14 (25.9)	8 (13.3)	13 (23.2)	9 (16.1)	44 (19.5)
Perampanel	9 (16.7)	4 (6.7)	13 (23.2)	10 (17.9)	36 (15.9)
Topiramate	8 (14.8)	10 (16.7)	5 (8.9)	10 (17.9)	33 (14.6)
Zonisamide	6 (11.1)	14 (23.3)	6 (10.7)	5 (8.9)	31 (13.7)

Abbreviations: ASM, antiseizure medication; b.i.d., twice daily; PSL, padsevonil; SD, standard deviation; VNS, vagus nerve stimulation.

^a^ Includes the following seizure types: focal aware with motor symptoms, focal impaired awareness, focal to bilateral tonic–clonic.

^b^ Includes previous ASMs and ASMs taken at trial entry.

#### 3.2.2 Efficacy

**FIGURE 2:**
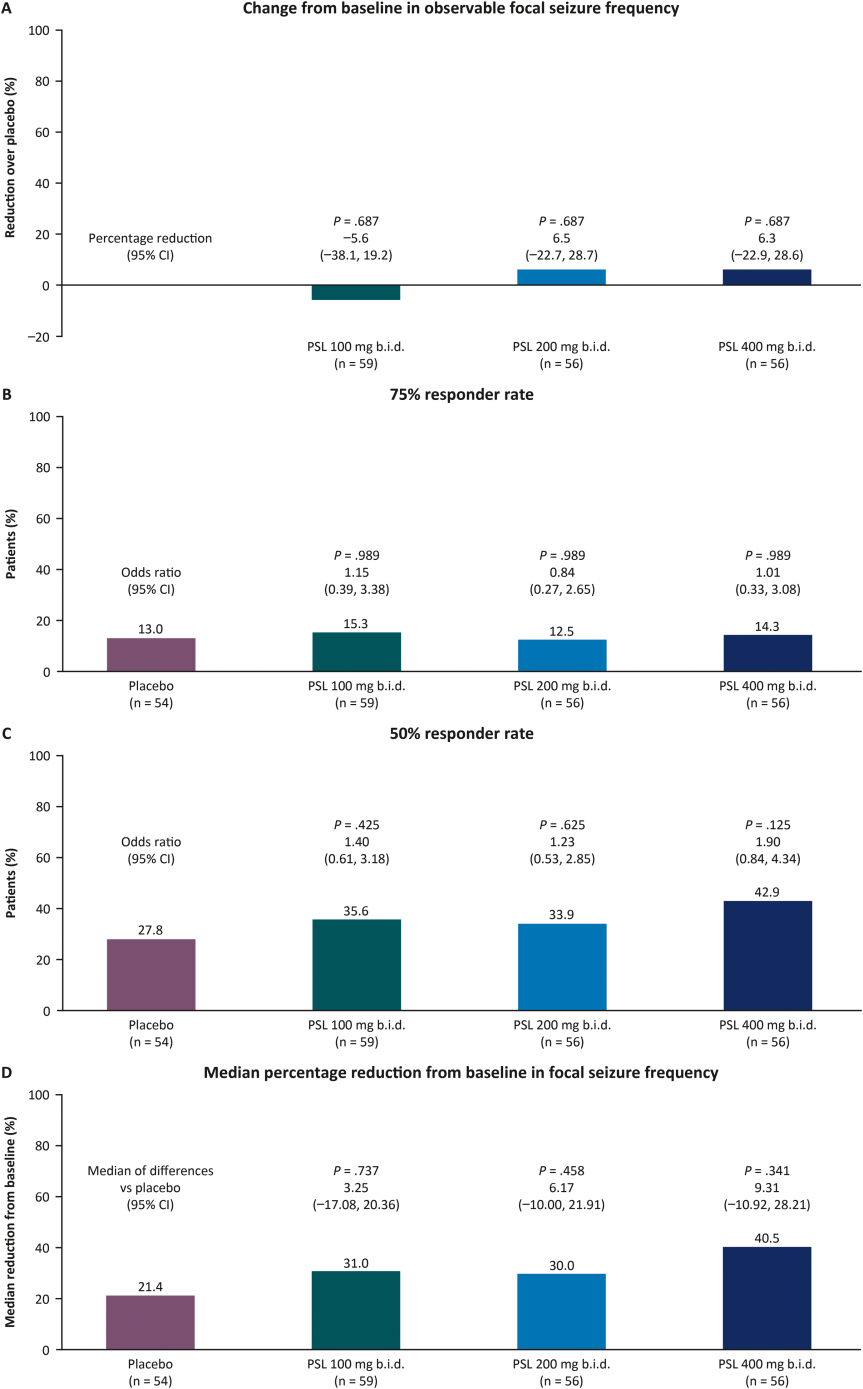
Efficacy outcomes of patients randomized to placebo or PSL by randomized dose group for observable focal seizures over the 12‐week maintenance period in the phase 3 efficacy trial (EP0092; full analysis set): (A) change from baseline in log‐transformed observable focal seizure frequency as percentage reductions over placebo; (B) 75% responder rate; (C) 50% responder rate; (D) median percentage reduction from baseline in focal seizures per 28 days. ASM, antiseizure medication; b.i.d., twice daily; PSL, padsevonil.

Two hundred and twenty‐six patients had baseline and postbaseline seizure frequency data and were included in efficacy assessments. The least squares mean change in log‐transformed observable focal seizure frequency was −0.35, −0.47, and −0.47 with PSL 100, 200, and 400 mg b.i.d. versus −0.41 with placebo. In patients on PSL 100, 200, 400 mg b.i.d., percentage reductions over placebo were −5.6% (*p* = 0.687), 6.5% (*p* = 0.687), and 6.3% (*p* = 0.687), respectively (Figure 2A), and 75% responder rates (and adjusted *p*‐values for odds ratios of PSL versus placebo) were not significantly different for any PSL dose group (15.3% [*p* = 0.989], 12.5% [*p* = 0.989], and 14.3% [*p* = 0.989], respectively) versus placebo (13.0%) (Figure 2B). 50% responder rates ranged from 33.9% to 42.9% in PSL dose groups versus 27.8% with placebo (Figure 2C). Median percentage reductions from baseline in observable focal seizure frequency per 28 days ranged from 30.0% to 40.5% with PSL versus 21.4% with placebo (Figure 2D). Among patients who completed the maintenance period, 2.3%, 6.8%, and 8.3% of patients on PSL 100 (*n* = 44), 200 (*n* = 44), and 400 mg b.i.d. (*n* = 36), respectively, versus 4.3% on placebo (*n* = 46) were free from all seizures.

#### 3.2.3 Tolerability

The overall incidence of TEAEs was numerically higher across PSL dose groups (range 78.9%–83.1%) than in the placebo group (67.3%) (Table 4; SS). More patients on PSL than placebo discontinued due to TEAEs, and the incidence generally increased with increasing PSL doses. The most common TEAEs with PSL (≥10% of all PSL patients) were somnolence (27.8%), dizziness (23.9%), fatigue (14.8%), and headache (13.6%) (placebo: 3.6%, 7.3%, 7.3%, and 14.5%, respectively).

**TABLE 4:** Trial EP0092 incidence of overall TEAEs and most common TEAEs with onset during the treatment period (safety set)

	Placebo (*n* = 55)	PSL 100 mg b.i.d. (*n* = 60)	PSL 200 mg b.i.d. (*n* = 57)	PSL 400 mg b.i.d. (*n* = 59)	All PSL patients (*n* = 176)
Any TEAEs, *n* (%)	37 (67.3)	48 (80.0)	45 (78.9)	49 (83.1)	142 (80.7)
Drug‐related TEAEs	15 (27.3)	30 (50.0)	31 (54.4)	43 (72.9)	104 (59.1)
Serious TEAEs	3 (5.5)	0	1 (1.8)	5 (8.5)	6 (3.4)
Severe TEAEs	2 (3.6)	4 (6.7)	4 (7.0)	3 (5.1)	11 (6.3)
Discontinuations due to TEAEs	2 (3.6)	6 (10.0)	6 (10.5)	12 (20.3)	24 (13.6)
Deaths	0	0	0	0	0
TEAEs^a^ reported by ≥5% of all patients on placebo or PSL, *n* (%)					
Somnolence	2 (3.6)	10 (16.7)	19 (33.3)	20 (33.9)	49 (27.8)
Dizziness	4 (7.3)	14 (23.3)	10 (17.5)	18 (30.5)	42 (23.9)
Fatigue	4 (7.3)	5 (8.3)	7 (12.3)	14 (23.7)	26 (14.8)
Headache	8 (14.5)	10 (16.7)	9 (15.8)	5 (8.5)	24 (13.6)
Asthenia	2 (3.6)	6 (10.0)	3 (5.3)	2 (3.4)	11 (6.3)
Insomnia	0	1 (1.7)	5 (8.8)	4 (6.8)	10 (5.7)
Irritability	3 (5.5)	4 (6.7)	3 (5.3)	3 (5.1)	10 (5.7)
Memory impairment	1 (1.8)	0	3 (5.3)	6 (10.2)	9 (5.1)
Nasopharyngitis	4 (7.3)	1 (1.7)	4 (7.0)	1 (1.7)	6 (3.4)
Nausea	3 (5.5)	1 (1.7)	3 (5.3)	1 (1.7)	5 (2.8)
Contusion	3 (5.5)	1 (1.7)	2 (3.5)	1 (1.7)	4 (2.3)
Diarrhea	3 (5.5)	1 (1.7)	0	2 (3.4)	3 (1.7)

Abbreviations: b.i.d., twice daily; PSL, padsevonil; TEAE, treatment‐emergent adverse event.

^a^ Medical Dictionary for Regulatory Activities Version 22.1 Preferred Term.

The incidences of serious TEAEs were generally low, reported by 3.4% of all PSL patients compared with 5.5% of patients on placebo. During the treatment period, two drug‐related serious TEAEs were reported in patients on PSL; one patient reported muscular weakness during titration and stabilization (dose reduced, resolved), and one patient reported hyponatremia during maintenance (dose not changed, resolved). Overall, PSL was generally well tolerated, with no new safety signals identified, and no clinically relevant differences between treatment groups were observed for any changes from baseline for hematology, blood chemistry, urinary parameters, vital signs, electrocardiograms, and echocardiograms.

## 4 DISCUSSION

In both double‐blind placebo‐controlled trials, the primary outcomes (change from baseline in observable focal seizure frequency and 75% responder rate over the 12‐week maintenance period) did not reach statistical significance in any PSL dose group compared with placebo. Numerical improvements were observed for the PSL dose groups compared with placebo; however, these did not show a dose‐dependent response.

In the dose‐finding trial, almost all patients had at least four lifetime ASMs, while many (52.1%) had eight or more, with a high baseline seizure frequency (median of 11.0 observable focal seizures per 28 days) indicating that these were a very drug‐resistant patient population. In phase 2/3 trials assessing efficacy and tolerability of adjunctive ASMs under development, the 50% responder rate is a commonly used primary efficacy outcome. In both trials, the 75% responder rate was selected as a primary efficacy outcome because it was regarded as clinically more meaningful than the 50% responder rate, and an improvement of >75% seemed feasible given the premise of the greater efficacy of PSL based on nonclinical animal data and results from the proof‐of‐concept trial.^9^, ^15^ It can be difficult to achieve a 75% responder rate in such a difficult‐to‐treat population.^3^, ^16^

In the dose‐finding trial, a numerically higher response was observed for both primary efficacy outcomes in PSL patients who were not taking SV2A ligands at trial entry versus those who were. However, even without concomitant SV2A drug intake, the efficacy of PSL was modest, and 75% responder rates were not better than those observed in pivotal trials with other ASMs in populations of comparable epilepsy severity.^16^, ^17^, ^18^, ^19^ Although primary outcomes did not reach statistical significance, the percentages of seizure freedom for patients taking PSL 200 or 400 mg b.i.d. in the dose‐finding trial (8.2% and 6.9%, respectively) were generally comparable to other trials of approved ASMs including BRV, lacosamide, and perampanel.^16^, ^17^, ^19^

PSL was generally well tolerated, and the safety profile was as expected. The most commonly reported TEAEs of somnolence, dizziness, fatigue, and headache were also the most common in the phase 2a proof‐of‐concept trial.^9^ In the phase 3 efficacy trial, the incidences of discontinuations due to TEAEs were higher in the PSL dose groups compared with the placebo group and generally increased with increasing PSL doses.

For the PK analysis in the dose‐finding trial, there was an overlap in exposure among the PSL doses. However, PSL and the desmethyl metabolite exposure increased with increasing dose, and differentiation in exposure was observed between the PSL 50 mg b.i.d. and PSL 400 mg b.i.d. groups. As steady‐state trough concentrations of PSL are in line with previous observations,^9^ this implies that the lack of expected efficacy in the dose‐finding trial was not a result of lower than anticipated exposure to PSL.

Patients who completed either of the double‐blind trials had the opportunity to receive PSL in an open‐label extension trial (EP0093), which was conducted to assess long‐term safety, tolerability, and efficacy.^20^ PSL was generally well tolerated in 406 patients with up to 19.4 months of treatment, with a safety profile consistent with that observed in the double‐blind trials. No safety signal related to abnormal echocardiogram findings was detected following the 6‐month follow‐up echocardiogram results. Consistency of an anticonvulsant effect was demonstrated in some patients for the duration of the trial.

With careful consideration and after an exhaustive evaluation of efficacy and safety data from the dose‐finding trial, it was concluded that although there was a trend for improvement over placebo, nonsignificant anticonvulsant efficacy was observed, and the magnitude of the effect of PSL seen in these trials suggested that it would not likely offer a higher clinically meaningful level of efficacy than many already available anticonvulsive treatments.

While these findings suggest an anticonvulsive mechanism of action‐related effects of PSL, in principle their magnitude did not indicate sufficient differentiation from available SV2A active ASMs, which would lead to meaningful clinical benefit for patients warranting further development. The clinical program did not provide adequate and well‐controlled data demonstrating the efficacy of PSL that would have been required for market authorization. Therefore, the sponsor decided not to further pursue the development of PSL and that the phase 3 efficacy trial and open‐label extension trial should be terminated. Patients were tapered off PSL, and the last patient's last visit for the phase 3 efficacy trial was on September 28, 2020.

Data from the phase 3 efficacy trial confirmed the conclusions from the dose‐finding trial, which indicated that for the overall population of patients with drug‐resistant focal epilepsy, PSL would not be more beneficial compared with currently available chronic anticonvulsive treatments. Given its early termination, the power of the phase 3 efficacy trial was reduced. However, even if the trial had been completed with the full sample size, observing a significant improvement with PSL would have been unlikely.

In summary, although numerical improvements in the primary efficacy outcomes were observed for PSL dose groups compared with placebo, these did not show a dose‐dependent response. In both trials, the primary outcomes did not reach statistical significance in any PSL dose group compared with placebo. Overall, PSL was generally well tolerated, and the safety profile was as expected with no new safety signals identified.

## AUTHOR CONTRIBUTIONS

Michael Rademacher, Manuel Toledo, Wim Van Paesschen, Kore K. Liow, and Ivan G. Milanov were involved in the execution of the trial as study investigators. Maria‐Luise Esch, Nan Wang, Merran MacPherson, William J. Byrnes, Timothy D. C. Minh, Elizabeth Webster, and Konrad J. Werhahn were involved in the study design and/or execution, and analysis and interpretation of the data. All authors critically reviewed the manuscript and approved the final version for submission.

## CONFLICT OF INTEREST

Michael Rademacher has received consultancy fees for reviewing the clinical study reports of these trials and participation in investigator meetings from UCB Pharma. Manuel Toledo has received grants and consultation honoraria from Arvelle Therapeutics, BIAL, Eisai, Esteve, Exeltis, GW Pharmaceuticals, Sanofi, and UCB Pharma. Wim Van Paesschen has received consultancy fees for participation in advisory boards for UCB Pharma. Kore K. Liow has received research support from Acadia, Aquestive Therapeutics, Biogen, Cerevel Therapeutics, Eisai, Engage Therapeutics, Idorsia, LivaNova, NeuroDerm, Novartis, SK Life Science, UCB Pharma, and Xenon Pharmaceuticals. Ivan G. Milanov has no conflicts of interest to disclose. Nan Wang was an employee of UCB Pharma at the time this trial was conducted and is currently affiliated with TechData Service Company LLC. Elizabeth Webster was an employee of UCB Pharma at the time this trial was conducted. Maria‐Luise Esch, Merran MacPherson, William J. Byrnes, Timothy D. C. Minh, and Konrad J. Werhahn are salaried employees of UCB Pharma and receive stock or stock options from their employment. Funding for writing support was provided by UCB Pharma.

## ETHICS STATEMENT

We confirm that we have read the Journal's position on issues involved in ethical publication and affirm that this report is consistent with those guidelines.

## Supporting information


Appendix S1

## Data Availability

Underlying data from this manuscript may be requested by qualified researchers 6 months after product approval in the United States and/or Europe, or global development is discontinued, and 18 months after trial completion. Investigators may request access to anonymized individual patient‐level data and redacted trial documents, which may include: analysis‐ready datasets, study protocol, annotated case report form, statistical analysis plan, dataset specifications, and clinical study report. Before the use of the data, proposals need to be approved by an independent review panel at www.Vivli.org, and a signed data sharing agreement will need to be executed. All documents are available in English only, for a prespecified time, typically 12 months, on a password‐protected portal.
