# Supplementary material for: Efficacy and safety of adjunctive padsevonil in adults with drug‐resistant focal epilepsy: Results from two double‐blind, randomized, placebo‐controlled trials
Source: Epilepsia Open. 2022 Oct 22;7(4):758–70. doi: 10.1002/epi4.12656 (PMC9712475; doi:10.1002/epi4.12656)
Supplement: Supplementary file 1 — Appendix S1 [file EPI4-7-758-s001.pdf]

Efficacy and safety of adjunctive padsevonil in adults with drug-resistant focal epilepsy: Results from two double-blind, randomized, placebo-controlled trials

Appendix S1

Exclusion criteria in trials EP0091 and EP0092

Patients were excluded from either trial if they:

- Had previously been randomized in a PSL trial or were receiving another trial medication;
- Had a history of (or current) medical condition or current psychiatric disorder that would have compromised their safety or ability to participate;
- Had either:
    - More than two times the upper limit of normal (ULN) of any of the following: alanine aminotransferase (ALT), aspartate aminotransferase (AST), alkaline phosphatase (ALP); or
    - >ULN total bilirubin (≥1.5 times ULN total bilirubin if known Gilbert's syndrome);
- Had a lifetime history of suicide attempt;
- Had a history of chronic alcohol or drug abuse within the past 2 years;
- Signs suggesting rapidly progressing brain disorder or brain tumor;
- Had a terminal illness or serious infection;
- Had a clinically significant abnormality on electrocardiogram that increased the risks associated with participation;
    - QT interval corrected for heart rate using Bazett's formula (QTcB) or QT interval corrected for heart rate using Fridericia's formula (QTcF) interval >450 ms;

    - Bundle branch blocks and other conduction abnormalities that were clinically significant according to the Investigator and/or with a PR interval ≥220 ms, irregular rhythms other than sinus arrhythmia or occasional, rare supraventricular, or rare ventricular ectopic beats in the opinion of the Investigator, or T-wave configurations that were not of sufficient quality for assessing QT interval duration;
    - A history of unexplained syncope or a family history of sudden death because of long QT syndrome;
- Had an abnormality on electrocardiogram at the screening visit as assessed by central reader that is accompanied by clinical symptoms or a ≥Grade 2*/moderate severity abnormality or a history of rheumatic heart disease or other known valvular abnormalities;
- Had a history of or signs of generalized or combined generalized and focal epilepsy;
- Had seizures that were uncountable (eg, because of clustering) on a regular basis 8 weeks before the screening visit and during the baseline period;
- Had isolated auras only;
- Had a current diagnosis of pseudo- or nonepileptic seizures;
- Had initiated an epilepsy dietary therapy within 3 months before the screening visit;
- Had vagus nerve stimulation, deep brain stimulation, Responsive Neurostimulator System, or other neurostimulation for epilepsy device:
    - Implanted and activated <1 year before enrollment; or
    - With stimulation parameters that had been stable for <3 months; or
    - With battery life of unit not anticipated to extend for duration of the trial;
- Were receiving carbamazepine, phenytoin, primidone, phenobarbital, or any strong inducer of cytochrome P450 (CYP) 3A4 liver enzymes;

- Previously had serious side effects with drugs with SV2A and γ-aminobutyric acid-A (GABA-A)-ergic mechanisms of action;
- Had a known hypersensitivity to any components of PSL formulation;
- Were taking any strong inducers or inhibitors of the cytochrome P450 (CYP) 3A4 or 2C19 pathway for 2 weeks before the baseline visit;
- Had been taking vigabatrin for <2 years at trial entry;
- Had been taking vigabatrin for ≥2 years without documented normal visual fields following ≥2 years of intake;
- Had a history of vigabatrin treatment and did not have a visual perimetry test ≥6 months following conclusion of treatment or the results of the visual perimetry test showed either a damage or a visual field defect associated with one of the following two conditions:
    - There was a change from a visual field test done at some point while the patient was taking vigabatrin; or
    - There was a change from a visual field test done within weeks after stopping vigabatrin administration;
- Had been taking felbamate for <12 months and/or had no appropriate laboratory tests showing no indication of aplastic anemia or hepatic failure;
- Had been taking retigabine for <4 years;
- Were regularly taking GABA-A-ergic drugs: agonists (ie, barbiturates) or receptor positive allosteric modulators (ie, benzodiazepines or nonbenzodiazepines); excluding as needed (prn) intake of GABA-A-ergic ASMs less than three times per week for emergencies.

- Patients were also excluded if in the 6 months before the screening visit, they had suicidal ideation, a cerebrovascular accident (including transient ischemic attack), or status

epilepticus, or had resective surgery for epilepsy (or plans for such surgery during the trial).

- Women were excluded if they were breastfeeding or pregnant, or planned to become pregnant.

Appendix S2

Full details of the statistical analyses

Change from baseline in observable focal seizure frequency from baseline adjusted to a 28-day frequency was analyzed for the FAS using ANCOVA, with treatment group (each PSL dose group and placebo) as the main factor, log-transformed baseline seizure frequency as a continuous covariate, and baseline SV2A use (yes or no) and region (Europe or Non-Europe) as categorical factors. Percent reduction over placebo was re-transformed regarding the estimated differences in least squares mean of the ANCOVA.

The odds ratio (PSL group versus placebo) for the 75% and 50% responder rates during the 12-week maintenance period were based on a logistic regression model with categorical factors for treatment group (each PSL dose group referenced to the placebo group), region (Europe or Non-Europe), baseline SV2A use (yes or no), and log-transformed baseline seizure frequency as a continuous covariate.

Nominal *P*-values (not adjusted for multiplicity) are presented for the median percentage reduction of observable focal seizure frequency and 50% responder rates (*P*-values for odds ratios of PSL group versus placebo). Adjusted *P*-values are presented for percentage reductions over placebo in observable focal seizure frequency and 75% responder rates (adjusted *P*-values for odds ratios of PSL group versus placebo), and are from the Hochberg step-up procedure within SAS® Proc Multtest to control Type I error. For the primary

outcomes (percentage reductions over placebo in observable focal seizure frequency and 75% responder rates), statistical analysis of the odds ratio of PSL 50 mg b.i.d. dose group compared to placebo was provided only if the other three higher doses were significant. Log-transformation was based on natural logarithm. For analyses on log-transformed values, model estimates were back-transformed to anti-log space for table presentation.

Appendix S3

Analysis by SV2A ligand (BRV and/or LEV) use at trial entry

In patients who were recorded on the electronic Case Report Form (eCRF) to have been taking LEV or BRV at trial entry randomized to PSL 50 (n = 41), 100 (n = 44), 200 (n = 46), and 400 mg b.i.d. (n = 44), percentage reductions over placebo (n = 39) in log-transformed observable focal seizure frequency were 9.5%, 16.7%, 13.6%, and −10.0%, respectively. Least squares mean change in log-transformed observable focal seizure frequency was −0.40, −0.48, −0.44, and −0.20 with PSL 50, 100, 200, 400 mg b.i.d., respectively, versus −0.30 with placebo. 75% responder rates were 4.9%, 11.4%, 6.5%, and 9.1% with PSL 50, 100, 200, and 400 mg b.i.d., respectively, versus 2.6% with placebo.

In patients who were recorded on the eCRF as not taking LEV or BRV at trial entry randomized to PSL 50 (n = 39), 100 (n = 38), 200 (n = 35), and 400 mg b.i.d. (n = 37), percentage reductions over placebo (n = 42) in log-transformed observable focal seizure frequency were 23.5%, 20.7%, 24.7%, and 33.1%, respectively. Least squares mean change in log-transformed observable focal seizure frequency was −0.54, −0.50, −0.56, and −0.67 with PSL 50, 100, 200, 400 mg b.i.d., respectively, versus −0.27 with placebo. 75% responder rates were 23.1%, 13.2%, 17.1%, and 24.3% with PSL 50, 100, 200, and 400 mg b.i.d., respectively, versus 9.5% with placebo.

FIGURE S1 EP0091 trial design

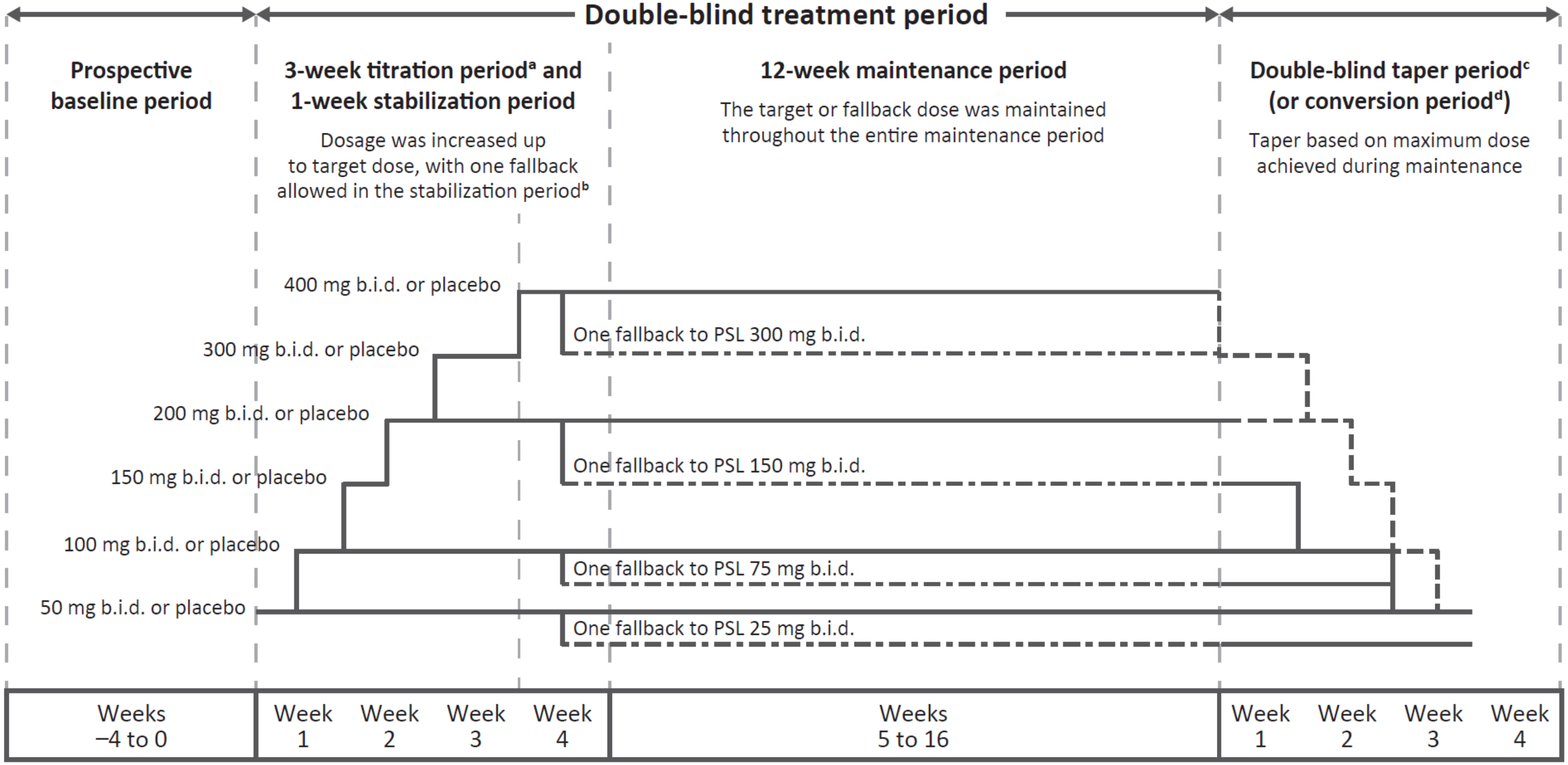

[a]Dosage was increased approximately every 3-7 days depending on treatment arm. A variation of ±1 day was allowed for each period of 3 or 4 days and a variation of ±3 days by week was acceptable, but the overall titration period variance in length was not to exceed 1 week. [b]At least 2 days before start of the 12-week maintenance period. [c]Taper steps could have varied in length by ±3 days per week. [d]In patients who entered the open-label trial (EP0093), treatments and doses were adjusted gradually in a blinded way to reach PSL 400 mg/day. b.i.d., twice daily; PSL, padsevonil.

FIGURE S2 EP0092 trial design

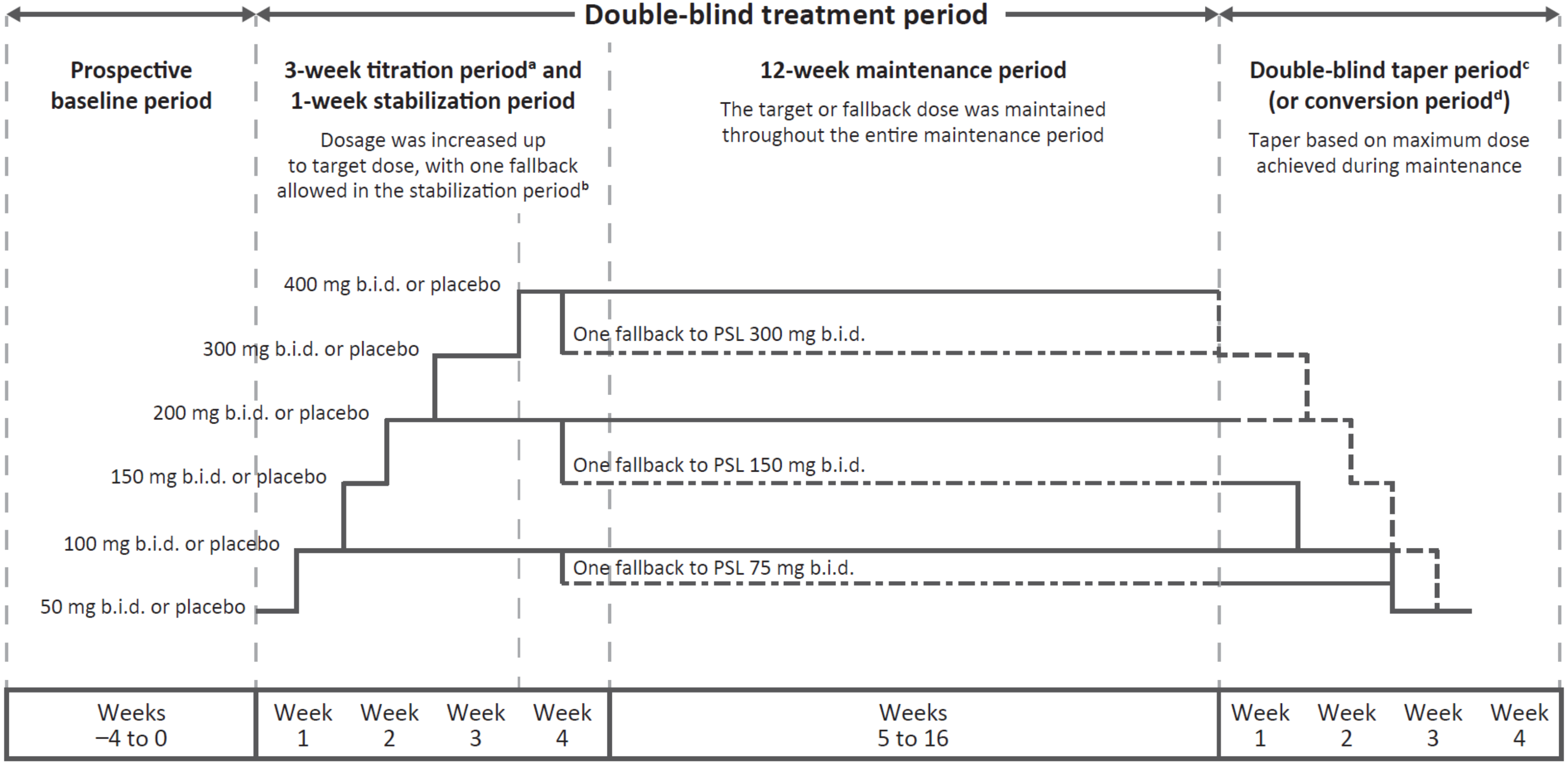

[a]Dosage was increased approximately every 3-7 days depending on treatment arm. A variation of ±1 day was allowed for each period of 3 or 4 days and a variation of ±2 days by week was acceptable, but the overall titration period variance in length was not to exceed 1 week. [b]At least 2 days before start of the 12-week maintenance period. [c]Taper steps could have varied in length by ±3 days per week. [d]In patients who entered the open-label trial (EP0093), treatments and doses were adjusted gradually in a blinded way to reach PSL 400 mg/day. b.i.d., twice daily; PSL, padsevonil.

FIGURE S3 Patient disposition and discontinuation in trial EP0091

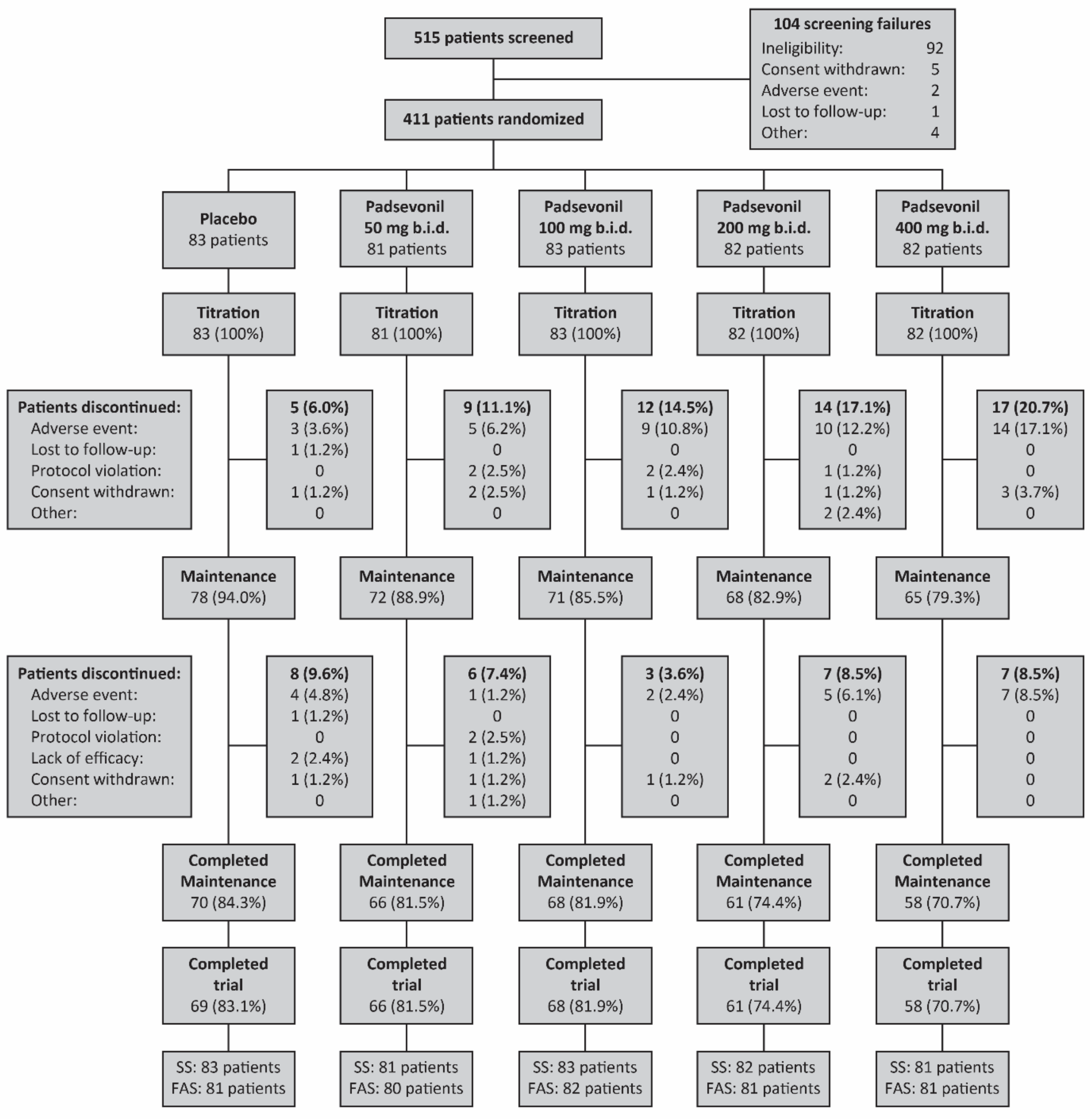

b.i.d., twice daily; FAS, Full Analysis Set; SS, Safety Set.

FIGURE S4 Geometric mean and 95% CI of (A) PSL and (B) the desmethyl metabolite trough blood concentrations by visit in trial EP0091 (PK-PPS)

A

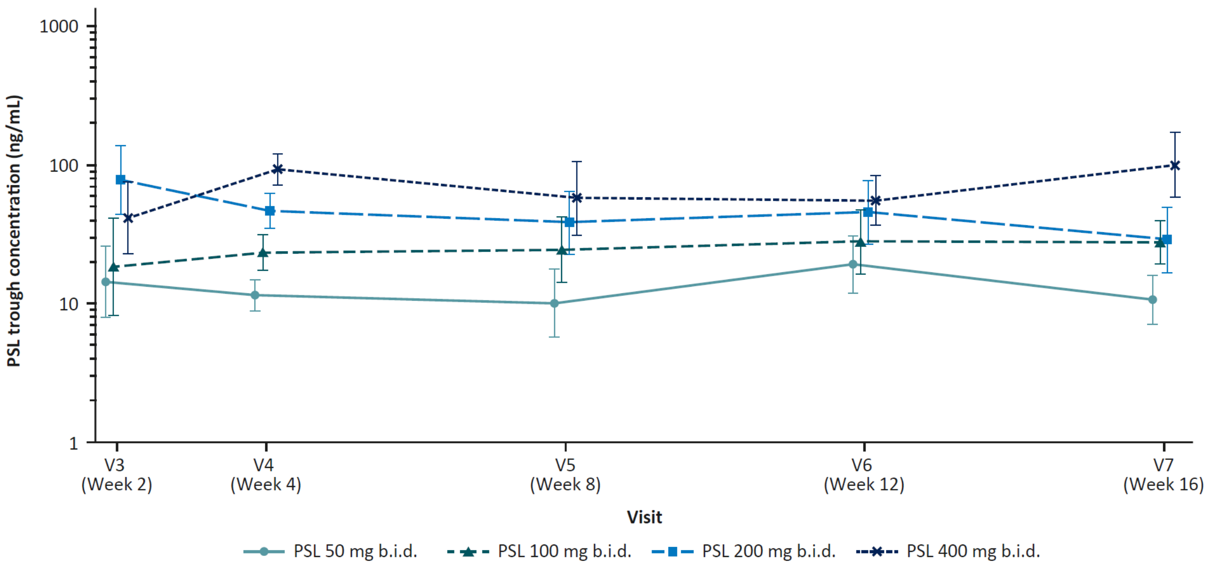

B

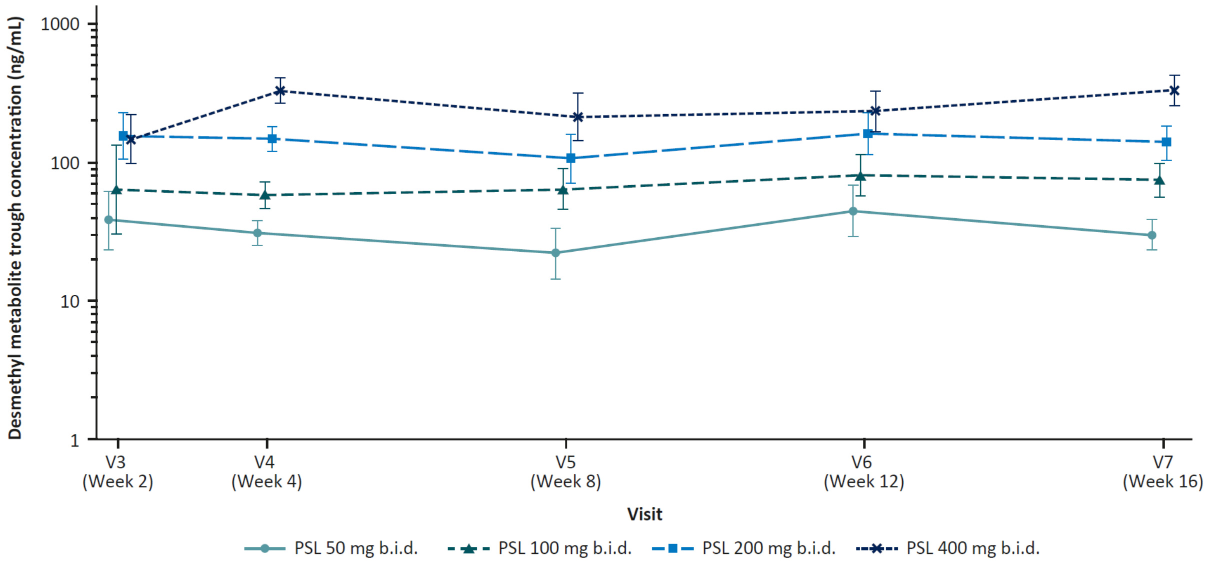

Trough concentrations 12-hours post dose were plotted.
b.i.d., twice daily; CI, confidence interval; PK-PPS, Pharmacokinetic Per-Protocol Set; PSL, padsevonil; V, visit.

FIGURE S5 Forest plot of LS mean ratios of the concentrations of concomitant ASM medications during the 12-week maintenance period versus baseline in trial EP0091 (ASM-PK-PPS)

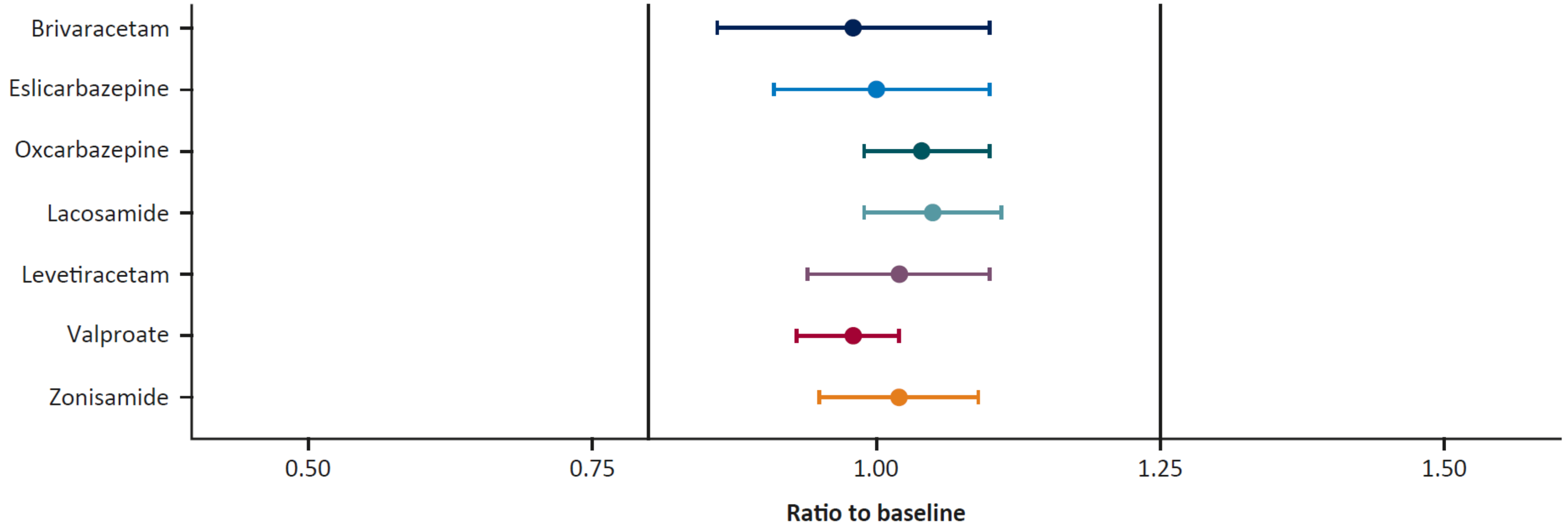

Only all maintenance for the PSL total group is presented. ASM, antiseizure medication; ASM-PK-PPS, Antiseizure Medication Pharmacokinetic Per-Protocol Set; LS, least squares.

FIGURE S6 Patient disposition and discontinuation in trial EP0092

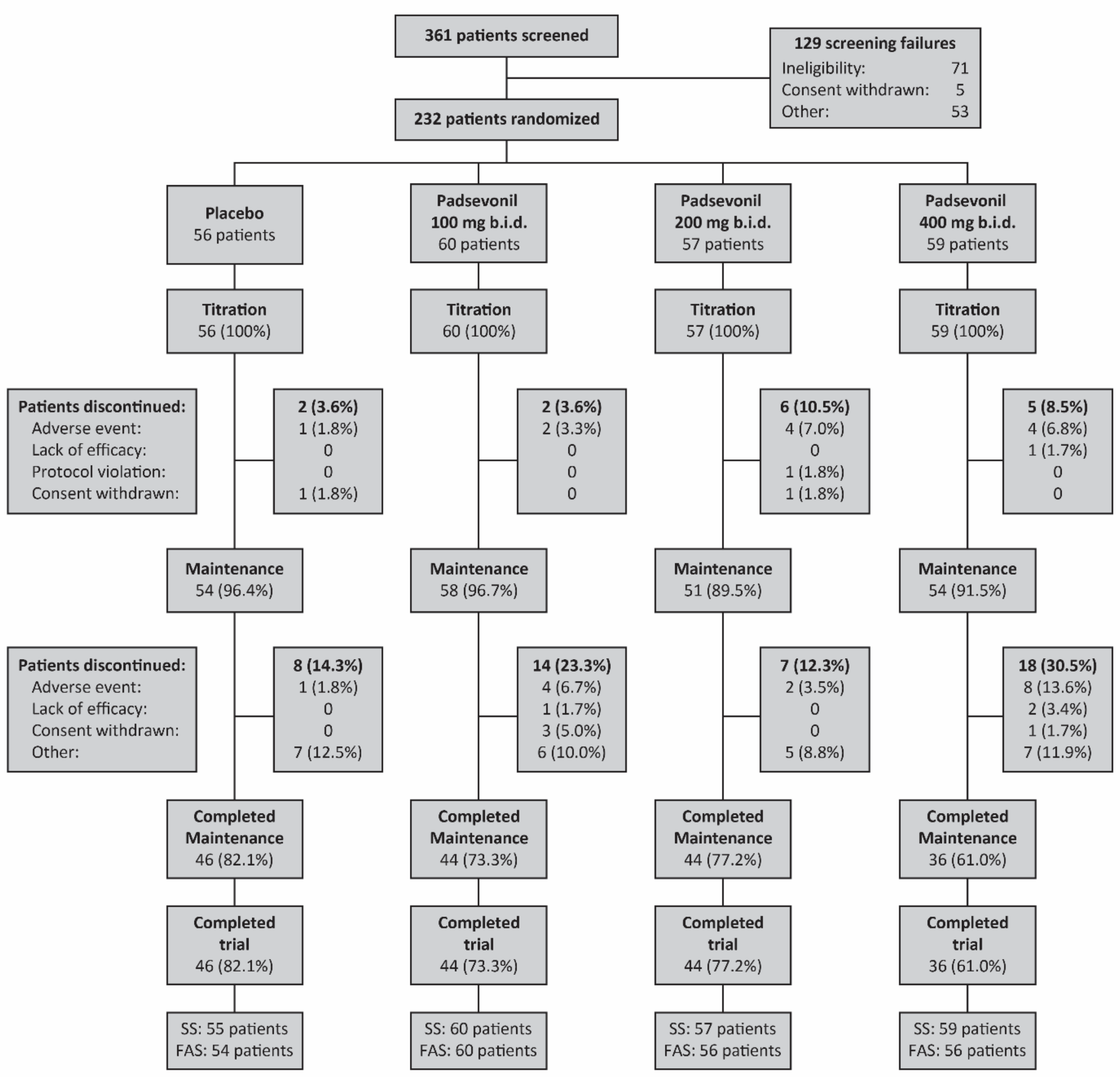

Discontinuations because of the coronavirus disease 2019 pandemic and the termination of the PSL development program are included in “other” reasons.
b.i.d., twice daily; FAS, Full Analysis Set; SS, Safety Set.

Appendix S4

Investigator appendix (EP0091)

The authors acknowledge the EP0091 trial investigators for their contributions to data acquisition: Bassel William Abou-Khalil, MD (Vanderbilt Epilepsy Center, Nashville, TN, USA); Sami Aboumatar, MD (Austin Epilepsy Care Center, Austin, TX, USA); Bola Adamolekun, MD (Wesley Neurology Clinic, Memphis, TN, USA); Umberto Aguglia, MD (Azienda Ospedaliera Bianchi-Melacrino-Morelli, Reggio Calabria, Italy); Naoki Akamatsu, MD (Fukuoka Sanno Hospital, Fukuoka, Japan); Heather Angus-Leppan, MD (Royal Free Hospital, London, UK); Yasukiyo Araki, MD (Shizuoka Institute of Epilepsy and Neurological Disorders, Shizuoka, Japan); Montserrat Asensio Asensio, MD (Hospital General Universitario de Alicante, Alicante, Spain); Ricardo Ayala, MD (Tallahassee Neurological Clinic, Tallahassee, FL, USA); Manny Bagary, MD (Barberry National Centre for Mental Health, Birmingham, UK); Hartmut Baier, MD (Die Weissenau, Zentrum Für Psychiatrie, Ravensburg, Germany); Michal Bajaček, MD (FORBELI s.r.o., Praha, Czech Republic); Attila Balogh, MD (Dél-pesti Centrumkórház, Országos Hematológiai és Infektológiai Intézet, Budapest, Hungary); Michal Bar, MD (Fakultní nemocnice Ostrava, Ostrava, Czech Republic); Petra Baum, MD (Klinik und Poliklinik für Neurologie, Universitätsklinikum Leipzig, Leipzig, Germany); Nerses Bebek, MD (Istanbul Faculty of Medicine, Istanbul University, Istanbul, Turkey); Samuel Berkovic, MD (Austin Health, Heidelberg, VIC, Australia); Arnaud Biraben, MD (Centre Hospitalier Universitaire de Rennes, Hôpital Pontchaillou, Rennes, France); Christian Brandt, MD (Bethel Epilepsy Center, Mara Hospital, Bielefeld, Germany); Deborah Briggs, MD (Seton Brain and Spine Institute, Austin, TX, USA); Dulce M. Campos Blanco, MD (Hospital Clínico Universitario de Valladolid, Valladolid, Spain); Laura Canafoglia, MD (Istituto Neurologico Carlo Besta, Milano, Italy); Teresa Cantisani, MD (Azienda Ospedaleria di Perugia, Perugia, Italy);

Patrick Carney, MD (Eastern Health Clinical Research Unit, Box Hill, VIC, Australia); Maria del Mar Carreño Martinez, MD (Hospital Clinic de Barcelona, Barcelona, Spain); Freddy Castro Farfan, MD (Grupo Médico Camino S.C. - Delegación Benito Juárez, Mexico City, Mexico); Jana Chamilova, MD (IN MEDIC s.r.o., Bardejov, Slovakia); Micaela Chatman, MD (Minnesota Epilepsy Group, St. Paul, MN, USA); Jocelyn Cheng, MD (New York University Langone Medical Center, New York, NY, USA); Roman Chwedorowicz, MD (Instytut Medycyny Wsi im. Witolda Chodzki, Lublin, Poland); Jean-Francois Clement, MD (Clinique Neuro Rive-Sud, Greenfield Park, QC, Canada); Arielle Crespel, MD (Centre Hospitalier Universitaire de Montpellier, Hôpital Gui De Chauliac, Montpellier, France); Giuseppe d'Orsi, MD (Azienda Ospedaliero Universitaria Ospedali Riuniti di Foggia, Foggia, Italy); Wendyl D'Souza, MD (St. Vincent's Hospital Melbourne, Fitzroy, VIC, Australia); María José Aguilar-Amat Prior, MD (Hospital Universitario La Paz, Madrid, Spain); Chantal Depondt, MD (Hôpital Erasme, Brussels, Belgium); Philippe Derambure, MD (Centre Hospitalier Universitaire de Lille, Hôpital Roger Salengro, Lille, France); Oguz Erdinc, MD (Eskisehir Osmangazi University Health Practice and Research Hospital, Eskisehir, Turkey); Jose Estévez, MD (Hospital Universitario Reina Sofia, Córdoba, Spain); María Mercedes Falip Centellas, MD (Hospital Universitario de Bellvitge, Barcelona, Spain); Silvana Franceschetti, MD (Istituto Neurologico Besta, Milan, Italy); Waldemar Fryze, MD (COPERNICUS Podmiot Leczn. Sp z o.o., Gdansk, Poland); Masafumi Fukuda, MD (Nishi-Niigata Chuo National Hospital, Niigata, Japan); Carlo Andrea Galimberti, MD (Istituto Neurologico Nazionale a Carattere Scientifico Fondazione Mondino, Pavia, Italy); Irene García Morales, MD (Hospital Clínico San Carlos, Madrid, Spain); Giedre Gelziniene, MD (Lietuvos sveikatos mokslu universiteto ligoninė, Kaunas, Lithuania); Plamen Georgiev, MD (MMA-MHAT Pleven Clinic for Neurology, Pleven, Bulgaria); Anna Giallonardo, MD (Policlinico Umberto I, Università La Sapienza, Roma, Italy); Antonio Gil-Nagel, MD

(Hospital Rúber Internacional, Madrid, Spain); Khalid Hamandi, MD (University Hospital of Wales, Cardiff, UK); Lyubomir Haralanov, MD (SHATCVD, National Heart Hospital, Sofia, Bulgaria); Jay Harvey, MD (University of Texas Southwestern Medical Center, Dallas, TX, USA); Edouard Hirsch, MD (Centre Hospitalier Universitaire de Strasbourg, Strasbourg, France); Koji Iida, MD (Hiroshima University Hospital, Hiroshima, Japan); Jan Ilkowski, MD (NZOZ NEURO-KARD Ilkowski i Partnerzy Sp. Partnerska Lekarzy, Poznan, Poland); Vicente Iváñez Mora, MD (Hospital Universitario La Paz, Madrid, Spain); Kousuke Kanemoto, MD (Aichi Medial University Hospital, Nagakute, Japan); Sasho Kastrev, MD (MHAT Puls, Blagoevgrad, Bulgaria); Mark Keezer, MD (Centre de Recherche du Centre Hospitalier de l'Université de Montréal, Montreal, QC, Canada); Anna Kelemen, MD (Országos Klinikai Idegtudományi Intézet, Budapest, Hungary); Christoph Kellinghaus, MD (Klinikum Osnabrück, Epilepsiezentrum Münster-Osnabrüeck, Osnabrück, Germany); Masako Kinoshita, MD (Utano National Hospital, Kyoto, Japan); Pavel Klein, MD (Mid-Atlantic Epilepsy and Sleep Center, Bethesda, MD, USA); Agata Klosek, MD (Twoja Przychodnia, Centrum Medyczne Nowa Sol, Nowa Sol, Poland); Susanne Knake, MD (Universitätsklinikum Giessen und Marburg GmbH, Marburg, Germany); Jitka Kocvarova, MD (Fakultní nemocnice u sv. Anny v Brne, Brno, Czech Republic); Gregory Krauss, MD (The Johns Hopkins Hospital, Baltimore, MD, USA); Yuichi Kubota, MD (TMG Asaka Medical Center, Asaka, Japan); Albrecht Kunze, MD (Universtitätsklinikum Jena, Jena, Germany); Marcelo Lancman, MD (Northeast Regional Epilepsy Group, Hackensack, NJ, USA); Kenneth Laxer, MD (California Pacific Medical Center, San Francisco, CA, USA); Martine Lemesle-Martin, MD (Centre Hospitalier Universitaire de Dijon, Dijon, France); Holger Lerche, MD (Universitätsklinikum Tübingen, Tübingen, Germany); Lubomir Lipovsky, MD (Neurologicka ambulancia MUDr. Eva Gasparova, Hlohovec, Slovakia); Alicja Lobinska, MD (NZOZ IGNIS Dr. Med. Alicja Lobinska, Swidnik, Poland); Elmer

Lopez Meza, MD (Neurociencias Estudios Clínicos S.C., Culiacán, Mexico); Ruta Mameniskiene, MD (Vilnius University Hospital Santaros Klinikos, Vilnius, Lithuania); Ainhoa Marinas Alejo, MD (Hospital de Cruces, Barakaldo, Spain); Meritxell Martínez Ferri, MD (Hospital Universitari MútuaTerrassa, Barcelona, Spain); Petr Marusic, MD (Fakultní nemocnice v Motole, Prague, Czech Republic); Dimitar Maslarov, MD (1st MHAT, Sofia, Bulgaria); Andreu Massot-Tarrús, MD (Hospital General Universitario Gregorio Marañón, Madrid, Spain); Sophie Mathais, MD (Centre Hospitalier Universitaire de Clermont-Ferrand, Hôpital Gabriel Montpied, Clermont-Ferrand, France); Oriano Mecarelli, MD (Università degli Studi di Roma La Sapienza, Roma, Italy); Andrea Menes, MD (Debreceni Egyetem Kenézy Gyula Egyetemi Kórház, Debrecen, Hungary); Nicola Biagio Mercuri, MD (Azienda Ospedaliera Universitaria Policlinico Tor Vergata, Università Roma Tor Vergata, Roma, Italy); Alfred Meurs, MD (UZ Gent, Gent, Belgium); Roberto Michelucci, MD (Università di Bologna, Ospedale Bellaria, Bologna, Italy); Seyed Mohammad Mirsattari, MD (London Health Sciences Center, London, ON, Canada); Gabriel Möddel, MD (Westfälische Wilhelms-Universität Münster, Münster, Germany); Rajiv Mohanraj, MD (Salford Royal NHS Foundation Trust, Manchester, UK); Eiji Nakagawa, MD (National Center of Neurology and Psychiatry, Tokyo, Japan); Vincent Navarro, MD (Hôpitaux Universitaires Pitié Salpetrière, Paris, France); Tomas Nezadal, MD (Institut neuropsychiatrické péče, Prague, Czech Republic); Hirotomo Ninomiya, MD (Itami City Hospital, Itami, Japan); Soheyl Noachtar, MD (Klinikum der Universität München, Campus Grosshadern, München, Germany); Terence O'Brien, MD (Alfred Health, Melbourne, VIC, Australia); Taku Ochiai, MD (Ochiai Brain Clinic, Saitama, Japan); Hemant Pandey, MD (MD First Research Chandler, Chandler, AZ, USA); Terry Peery, MD (Central Texas Neurology Consultants, Round Rock, TX, USA); Plamen Petkov, MD (Medical Center Vita 1 Ltd, Pleven, Bulgaria); Monica Puligheddu, MD (Azienda Ospedaliera Universitaria di Cagliari, Cagliari, Italy); Pier

Paolo Quarato, MD (Istituto Neurologico Mediterraneo Neuromed è un Istituto di Ricovero e Cura a Carattere Scientifico (IRCSS), Pozzilli, Italy); Samiya Rashid, MD (The University of Texas Health Science Center, San Antonio, TX, USA); David Reutens, MD (Royal Brisbane and Women's Hospital, Herston, QLD, Australia); Rodrigo Rocamora Zúñiga, MD (Hospital del Mar, Barcelona, Spain); Dominique Rosenberg, MD (Centre Hospitalier Universitaire de Clermont-Ferrand, Hôpital Gabriel Montpied, Clermont-Ferrand, France); Felix Rosenow, MD (Universitätsklinikum Frankfurt, Frankfurt am Main, Germany); Ahmed Sadek, MD (Research Institute of Orlando LLC, Orlando, FL, USA); Inder Sawhney, MD (Morriston Hospital, Swansea, UK); Andreas Schulze-Bonhage, MD (Universitätsklinikum Freiburg, Freiburg, Germany); Eric Segal, MD (Northeast Regional Epilepsy Group, Hackensack, NJ, USA); Udaya Seneviratne, MD (Monash Medical Centre, Monash Health, Clayton, VIC, Australia); Pedro Jesús Serrano Castro, MD (Hospital Regional Universitario de Málaga, Málaga, Spain); José Serratosa Fernández, MD (Hospital Universitario Fundación Jiménez Díaz, Madrid, Spain); Jerry Shih, MD (University of California, San Diego, La Jolla, CA, USA); Michael R. Sperling, MD (Comprehensive Epilepsy Center at Thomas Jefferson University, Philadelphia, PA, USA); Bernhard Steinhoff, MD (Kork Epilepsy Centre, Kehl-Kork, Germany); Stefan Stodieck, MD (Ev. Krankenhaus Alsterdorf gemeinnützige GmbH, Hamburg, Germany); Hans-Beatus Straub, MD (Epilepsie-Zentrum Berlin-Brandenburg, Berlin, Germany); Andrzej Szczudlik, MD (Krakowska Akademia Neurologii Sp. z o.o., Krakow, Poland); Marzena Tomaszewska, MD (Mazowieckie Centrum Badań Klinicznych s.c., Grodzisk Mazowiecki, Poland); Annelies Van Dycke, MD (AZ Sint-Jan Brugge-Oostende, Campus Sint Jan, Bruges, Belgium); Blanca Vazquez-Santana, MD (New York University Langone Medical Center, New York, NY, USA); Vicente Villanueva Haba, MD (Hospital Universitari i Politecnic La Fe, Valencia, Spain); Dana Vyskočilová, MD (Fakultní Thomayerova nemocnice, Prague, Czech Republic); Yvonne Weber, MD

(Universitätsklinikum Tübingen, Tübingen, Germany); Florian Weissinger, MD (Vivantes Humboldt-Klinikum, Berlin, Germany); Manori Wijayath, MD (Westmead Hospital, Westmead, NSW, Australia); Jaroslaw Wronka, MD (Clinical Research Center Sp. z o.o., Medic-R Sp. K., Poznan, Poland); Michael Xu, MD (OSF St. Francis Hospital, Peoria, IL, USA); Seher Naz Yeni, MD (Istanbul University-Cerrahpaşa Medical Faculty, Istanbul, Turkey); Christian Zentner, MD (Prince of Wales Hospital, Randwick, NSW, Australia); Tomasz Zielinski, MD (NZOZ Novo Med, Katowice, Poland).

Investigator appendix (EP0092)

The authors acknowledge the EP0092 trial investigators for their contributions to data acquisition: Yasukiyo Araki, MD (Shizuoka Institute of Epilepsy and Neurological Disorders, Shizuoka, Japan); Julia Aram, MD (Brighton and Sussex University Hospitals NHS Trust, Brighton, UK); Montserrat Asensio Asensio, MD (Hospital General Universitario de Alicante, Alicante, Spain); Michal Bajaček, MD (FORBELI s.r.o., Prague, Czech Republic); Edin Basagic, MD (Cantonal Hospital Dr. Irfan Ljubijankic, Bihać, Bosnia and Herzegovina); Silvio Basic, MD (University Hospital Dubrava, Zagreb, Croatia); Petra Baum, MD (Klinik und Poliklinik für Neurologie, Universitätsklinikum, Leipzig, Germany); Robert Beach, MD (State University of New York Upstate Medical University Hospital, Syracuse, NY, USA); Nerses Bebek, MD (Istanbul University Istanbul Medical Faculty, Istanbul, Turkey); Christoph Beier, MD (Odense University Hospital, Odense, Denmark); Elinor Ben-Menachem, MD (Sahlgrenska Universitetssjukhuset, Göteborg, Sweden); Meriem Bensalem-Owen, MD (University of Kentucky, Lexington, KY, USA); Samuel Berkovic, MD (Austin Health, Heidelberg, VIC, Australia); Yasemin Bicer Gomceli, MD (SBU Antalya Training and Research Hospital, Antalya, Turkey); Larry Blankenship, MD (Community Clinical Research Center, Anderson, IN, USA); Guillermo Blanco, MD (Vital

Pharma Research, Inc, Hialeah, FL, USA); Alexis Boro, MD (Montefiore Medical Center/Albert Einstein College of Medicine, Bronx, NY, USA); Plamen Bozhinov, MD (University Hospital Pleven, Pleven, Hungary); Christian Brandt, MD (Bethel Epilepsy Center, Mara Hospital, Bielefeld, Germany); Anna Brzozowska Jaśkiewicz, MD (ETG Lublin, Lublin, Poland); Marlene Bultemeyer, MD (Fort Wayne Neurological Center, Fort Wayne, IN, USA); Laura Canafoglia, MD (Istituto Neurologico Carlo Besta, Milano, Italy); Patrick Carney, MD (Box Hill Hospital, Box Hill, Australia); Jana Chamilova, MD (IN MEDIC s.r.o., Bardejov, Slovakia); Francine Chassoux, MD (Centre Hospitalier Sainte-Anne, Paris, France); Justyna Chojdak-Lukasiewicz, MD (Vistamed, Wroclaw, Poland); Jakob Christensen, MD (Aarhus University Hospital, Aarhus, Denmark); Paulo Coelho, MD (Hospital Pedro Hispano-Unidade Local de Saúde de Matosinhos, Matosinhos, Portugal); Daniel Costello, MD (Cork University Hospital, Cork, Ireland); Piotr Czapinski, MD (NZOZ Centrum Leczenia Padaczki i Migreny, Krakow, Poland); Maria Dabrowska, MD (ETG Zgierz, Zgierz, Poland); Adrien Didelot, MD (Hôpital Saint Joseph Saint Luc, Lyon, France); Colin P. Doherty, MD (St. James Hospital, Dublin, Ireland); Wendyl D'Souza, MD (St. Vincent's Hospital Melbourne, Fitzroy, VIC, Australia); Barbara Dworetzky, MD (Harvard Medical School, Brigham and Women's Hospital, Boston, MA, USA); Riem El Tahry, MD (Centre de référence pour l'épilepsie réfractaire, Cliniques universitaires Saint-Luc, Bruxelles, Belgium); María Mercedes Falip Centellas, MD (Hospital Universitario de Bellvitge, Barcelona, Spain); Susana Ferrao Santos, MD (Centre de référence pour l'épilepsie réfractaire, Cliniques universitaires Saint-Luc, Bruxelles, Belgium); Evan Fertig, MD (Providence Neurological Specialties West, Portland, OR, USA); Nathan Fountain, MD (University of Virginia, Charlottesville, VA, USA); Masami Fujii, MD (Yamaguchi Prefectural Grand Medical Center, Hofu, Japan); Ayataka Fujimoto, MD (Seirei Hamamatsu General Hospital, Comprehensive Epilepsy Center, Hamamatsu, Japan); Kyriakos Garganis,

MD (Saint Luke's Hospital, Thessaloniki, Greece); Jacek Gawlowicz, MD (Clinical Best Solutions, Lublin, Poland); Ksenija Gebauer Bukurov, MD (Clinical Center of Vojvodina, Novi Sad, Serbia); Plamen Georgiev, MD (MMA-MHAT Pleven Clinic for Neurology, Pleven, Bulgaria); Ana Maria Ghitoiu, MD (Med Life, Bucuresti, Romania); Arthur Grant, MD (State University of New York Downstate Medical Center, Brooklyn, NY, USA); Hans Grebe, MD (Hospital São Sebastião, Centro Hospitalar de Entre o Douro e Vouga, Santa Maria da Feira, Portugal); Katrin Gross-Paju, MD (Astra Team Clinic, Tallinn, Estonia); Candan Gürses, MD (Koc University Hospital, Istanbul, Turkey); Sulev Haldre, MD (Tartu University Hospital, Tartu, Estonia); Satoru Hiroshima, MD (Asahikawa Medical University Hospital, Asahikawa, Japan); Edouard Hirsch, MD (Centre Hospitalier Universitaire de Strasbourg, Strasbourg, France); Koji Iida, MD (Hiroshima University Hospital, Hiroshima, Japan); Jozsef Janszky, MD (Pécsi Tudományegyetem Klinikai Központ, Pécs, Hungary); Helena Berit Jonsson Gauffin, MD (Universitetssjukhuset Linköping, Linköping, Sweden); Kousuke Kanemoto, MD (Aichi Medial University Hospital, Nagakute, Japan); Omer Karadas, MD (Gülhane Training and Research Hospital, Ankara, Turkey); Sasho Kastrev, MD (MHAT Puls, Blagoevgrad, Bulgaria); Dimitrios Kazis, MD (G. Papanikolaou Hospital, Thessaloniki, Greece); Anna Kelemen, MD (Országos Klinikai Idegtudományi Intézet, Budapest, Hungary); Vasilios Kimiskidis, MD (Aristotle University of Thessaloniki, AHEPA University Hospital, Thessaloniki, Greece); Demet Kinay, MD (Türkiye Cumhuriyeti Sağlik Bakanliği Okmeydani Education and Research Hospital, Istanbul, Turkey); Masako Kinoshita, MD (Utano National Hospital, Kyoto, Japan); Pavel Klein, MD (Mid-Atlantic Epilepsy and Sleep Center, Bethesda, MD, USA); Agata Klosek, MD (Twoja Przychodnia, Centrum Medyczne Nowa Sol, Nowa Sol, Poland); Inge Klupka-Saric, MD (University Clinical Hospital Mostar, Mostar, Bosnia and Herzegovina); Jitka Kocvarova, MD (Fakultní nemocnice u sv. Anny v Brne, Brno, Czech Republic); Shinji Kondo, MD (Sanin Rosai

Hospital, Tottori, Japan); Mohamad Koubeissi, MD (George Washington University Medical Faculty Associates, Washington, DC, USA); Gregory Krauss, MD (The Johns Hopkins Hospital, Baltimore, MD, USA); Suparna Krishnaiengar, MD (University of Florida, Jacksonville, FL, USA); Yuichi Kubota, MD (TMG Asaka Medical Center, Asaka, Japan); Albrecht Kunze, MD (Universtitätsklinikum Jena, Jena, Germany); Ruben Kuzniecky, MD (Northwell Health, Lenox Hill, New York, NY, USA); Athanassios P. Kyritsis, MD (University Hospital of Ioannina, Ioannina, Greece); David Labiner, MD (University of Arizona Health Sciences, Tucson, AZ, USA); Ilo Leppik, MD (MINCEP Epilepsy Care, Minneapolis, MN, USA); Ande Lindmae, MD (North ESTONIA Medical Centre, Tallinn, Estonia); Taketoshi Maehara, MD (Tokyo Medical and Dental University, Tokyo, Japan); Petr Marusic, MD (Fakultní nemocnice v Motole, Prague, Czech Republic); Anatol Mickielewicz, MD (Centrum Medyczne Pratia Warszawa, Warsaw, Poland); Ioana Mindruta, MD (Sapiens Medical Center, Bucuresti, Romania); Rajiv Mohanraj, MD (Salford Royal NHS Foundation Trust, Manchester, UK); David Moore, MD (McFarland Clinic PC, Ames, IA, USA); Valéria Nagy, MD (Debreceni Egyetem Kenézy Gyula Egyetemi Kórház, Debrecen, Hungary); Hidetoshi Nakamoto, MD (TMG Asaka Medical Center, Asaka, Japan); Armin Nikpour, MD (University of Sydney, Royal Prince Alfred Hospital, Camperdown, Australia); Hirotomo Ninomiya, MD (Itami City Hospital, Itami, Japan); Terence O'Brien, MD (Alfred Health, Melbourne, VIC, Australia); Taku Ochiai, MD (Ochiai Neurological Clinic, Saitama, Japan); Antonio Oliveros Cid, MD (Hospital Viamed Montecanal, Zaragoza, Spain); Tomonori Ono, MD (National Hospital Organization Nagasaki Medical Center, Nagasaki, Japan); Ilona Palka-Kisielowska, MD (Centrum Medyczne Pratia Katowice, Katowice, Poland); Hemant Pandey, MD (MD First Research Chandler, Chandler, AZ, USA); Sima Patel, MD (MINCEP Epilepsy Care, Minneapolis, MN, USA); Jukka Peltola, MD (Tampere University Hospital, Tampere, Finland); Shankar Perumal, MD (Novant

Health Presbyterian Medical Center, Charlotte, NC, USA); Željka Petelin Gadže, MD (Klinički bolnički centar Zagreb, Zagreb, Croatia); Robert Powell, MD (Morriston Hospital, Swansea, UK); Rodrigo Rocamora Zúñiga, MD (Hospital del Mar, Barcelona, Spain); Joanne Rogin, MD (Minneapolis Clinic of Neurology Ltd, Golden Valley, MN, USA); Felix Rosenow, MD (Universitätsklinikum Frankfurt, Frankfurt am Main, Germany); Ahmed Sadek, MD (Research Institute of Orlando LLC, Orlando, FL, USA); Yosuke Sato, MD (Showa University Hospital, Tokyo, Japan); Mona Sazgar, MD (University of California, Irvine Medical Center, Orange, CA, USA); Hae Won Shin, MD (North Carolina Translational and Clinical Sciences [NC TraCS] Institute, Chapel Hill, NC, USA); Hideaki Shiraishi, MD (Hokkaido University Hospital, Sapporo, Japan); Jaysingh Singh, MD (Ohio State University, Columbus, OH, USA); Jana Slonkova, MD (Cerebrovaskularni poradna s.r.o., Ostrava, Czech Republic); Dzevdet Smajlovic, MD (University Clinical Centre Tuzla, Tuzla, Bosnia and Herzegovina); Dragoslav Sokic, MD (Clinical Center of Serbia, Belgrade, Serbia); Michael R. Sperling, MD (Comprehensive Epilepsy Center at Thomas Jefferson University, Philadelphia, PA, USA); Mariusz Stasiolek, MD (Uniwersytecki Szpital Kliniczny nr 1 im. N. Barlickiego UM w Łodzi, Łódź, Poland); Hans-Beatus Straub, MD (Epilepsie-Zentrum Berlin-Brandenburg, Berlin, Germany); Hidenori Sugano, MD (Juntendo University Hospital, Tokyo, Japan); Enra Suljic, MD (Clinical Center University of Sarajevo, Sarajevo, Bosnia and Herzegovina); Yasuhiro Suzuki, MD (Osaka Women's and Children's Hospital, Osaka, Japan); Torleiv Svendsen, MD (Spesialsykehuset for epilepsi [SSE], Sandvika, Norway); Andrzej Szczudlik, MD (Krakowska Akademia Neurologii Sp. z o.o., Krakow, Poland); Ishitsu Takateru, MD (Kumamoto-Ezuko Medical Center for The Severely Disabled, Kumamoto, Japan); Christian Tilz, MD (Krankenhaus Barmherzige Brüder Regensburg, Regensburg, Germany); Rafael Toledano Delgado, MD (Hospital Universitario Ramón y Cajal, Madrid, Spain); Sanja Tomasović, MD (Klinička bolnica Sveti Duh, Zagreb,

Croatia); Tsuyoshi Torii, MD (National Hospital Organization Kure Medical Center, Kure, Japan); Naohiro Tsuyuguchi, MD (Kindai University Hospital, Osaka, Japan); Sibel Velioglu, MD (Karadeniz Technical University, Farabi Hospital, Trabzon, Turkey); Vicente Villanueva Haba, MD (Hospital Universitari i Politecnic La Fe, Valencia, Spain); David Vossler, MD (UW Valley Medical Center, Renton, WA, USA); Pascal Vrielynck, MD (Centre Hospitalier Neurologique William Lennox, Ottignies, Belgium); Florian Weissinger, MD (Vivantes Humboldt-Klinikum, Berlin, Germany); Ewa Wierzchowska-Cioch, MD (ETG Zamość, Zamość, Poland); Sanjay Yathiraj, MD (Nova Clinical Research, LLC, Bradenton, FL, USA); Iris Zavoreo, MD (Klinički bolnički centar Sestre milosrdnice, Zagreb, Croatia); Tomasz Zielinski, MD (NZOZ Novo Med, Katowice, Poland); Salman Zubair, MD (Hightower Clinical, Oklahoma City, OK, USA).

Investigator appendix (EP0093)

The authors acknowledge the EP0093 trial investigators for their contributions to data acquisition: Bassel William Abou-Khalil, MD (Vanderbilt Epilepsy Center, Nashville, TN, USA); Sami Aboumatar, MD (Austin Epilepsy Care Center, Austin, TX, USA); Bola Adamolekun, MD (Wesley Neurology Clinic, Memphis, TN, USA); Yasukiyo Araki, MD (Shizuoka Institute of Epilepsy and Neurological Disorders, Shizuoka, Japan); Julia Aram, MD (Brighton and Sussex University Hospitals NHS Trust, Brighton, UK); Montserrat Asensio Asensio, MD (Hospital General Universitario de Alicante, Alicante, Spain); Manny Bagary, MD (Barberry National Centre for Mental Health, Birmingham, UK); Michal Bajaček, MD (FORBELI s.r.o., Praha, Czech Republic); Attila Balogh, MD (Dél-pesti Centrumkórház, Országos Hematológiai és Infektológiai Intézet, Budapest, Hungary); Michal Bar, MD (Fakultní nemocnice Ostrava, Ostrava, Czech Republic); Petra Baum, MD (Klinik und Poliklinik für Neurologie, Universitätsklinikum Leipzig, Leipzig, Germany); Robert Beach, MD (State University of New York Upstate Medical University Hospital, Syracuse,

NY, USA); Nerses Bebek, MD (Istanbul University Istanbul Medical Faculty, Istanbul, Turkey); Christoph Beier, MD (Odense University Hospital, Odense, Denmark); Meriem Bensalem-Owen, MD (University of Kentucky, Lexington, KY, USA); Samuel Berkovic, MD (Austin Health, Heidelberg, VIC, Australia); Arnaud Biraben, MD (Centre Hospitalier Universitaire de Rennes, Hôpital Pontchaillou, Rennes, France); Alexis Boro, MD (Montefiore Medical Center/Albert Einstein College of Medicine, Bronx, NY, USA); Plamen Bozhinov, MD (University Hospital Pleven, Pleven, Hungary); Christian Brandt, MD (Bethel Epilepsy Center, Mara Hospital, Bielefeld, Germany); Deborah Briggs, MD (Seton Brain and Spine Institute, Austin, TX, USA); Marlene Bultemeyer, MD (Fort Wayne Neurological Center, Fort Wayne, IN, USA); Dulce M. Campos Blanco, MD (Hospital Clínico Universitario de Valladolid, Valladolid, Spain); Laura Canafoglia, MD (Istituto Neurologico Carlo Besta, Milano, Italy); Patrick Carney, MD (Eastern Health Clinical Research Unit, Box Hill, VIC, Australia); Maria del Mar Carreño Martinez, MD (Hospital Clinic de Barcelona, Barcelona, Spain); Freddy Castro Farfan, MD (Grupo Médico Camino S.C. - Delegación Benito Juárez, Mexico City, Mexico); Jana Chamilova, MD (IN MEDIC s.r.o., Bardejov, Slovakia); Micaela Chatman, MD (Minnesota Epilepsy Group, St. Paul, MN, USA); Jakob Christensen, MD (Aarhus University Hospital, Aarhus, Denmark); Roman Chwedorowicz, MD (Instytut Medycyny Wsi im. Witolda Chodzki, Lublin, Poland); Jean Francois Clement, MD (Clinique Neuro Rive-Sud, Greenfield Park, QC, Canada); Daniel Costello, MD (Cork University Hospital, Cork, Ireland); Arielle Crespel, MD (Centre Hospitalier Universitaire de Montpellier, Hôpital Gui De Chauliac, Montpellier, France); Piotr Czapinski, MD (NZOZ Centrum Leczenia Padaczki i Migreny, Krakow, Poland); Giuseppe d’Orsi, MD (Azienda Ospedaliero Universitaria Ospedali Riuniti di Foggia, Foggia, Italy); Wendyl D’Souza, MD (St. Vincent’s Hospital Melbourne, Fitzroy, VIC, Australia); Chantal Depondt, MD (Hôpital Erasme, Brussels, Belgium); Philippe Derambure, MD (Centre Hospitalier Universitaire de

Lille, Hôpital Roger Salengro, Lille, France); Barbara Dworetzky, MD (Harvard Medical School, Brigham and Women's Hospital, Boston, MA, USA); Oguz Erdinc, MD (Eskisehir Osmangazi University Health Practice and Research Hospital, Eskisehir, Turkey); Jose Estévez, MD (Hospital Universitario Reina Sofia, Córdoba, Spain); María Mercedes Falip Centellas, MD (Hospital Universitario de Bellvitge, Barcelona, Spain); Waldemar Fryze, MD (COPERNICUS Podmiot Leczn. Sp z o.o., Gdansk, Poland); Masami Fujii, MD (Yamaguchi Prefectural Grand Medical Center, Hofu, Japan); Ayataka Fujimoto, MD (Seirei Hamamatsu General Hospital, Comprehensive Epilepsy Center, Hamamatsu, Japan); Masafumi Fukuda, MD (Nishi-Niigata Chuo National Hospital, Niigata, Japan); Carlo Andrea Galimberti, MD (Istituto Neurologico Nazionale a Carattere Scientifico Fondazione Mondino, Pavia, Italy); Irene García Morales, MD (Hospital Clínico San Carlos, Madrid, Spain); Kyriakos Garganis, MD (Saint Luke's Hospital, Thessaloniki, Greece); Jacek Gawlowicz, MD (Clinical Best Solutions, Lublin, Poland); Giedre Gelziniene, MD (Lietuvos sveikatos mokslu universiteto ligoninė, Kaunas, Lithuania); Plamen Georgiev, MD (MMA-MHAT Pleven Clinic for Neurology, Pleven, Bulgaria); Ana Maria Ghitoiu, MD (Med Life, Bucuresti, Romania); Anna Giallonardo, MD (Policlinico Umberto I, Università La Sapienza, Roma, Italy); Antonio Gil-Nagel, MD (Hospital Rúber Internacional, Madrid, Spain); Katrin Gross-Paju, MD (Astra Team Clinic, Tallinn, Estonia); Candan Gürses, MD (Koc University Hospital, Istanbul, Turkey); Koichi Hagiwara, MD (Fukuoka Sanno Hospital, Fukuoka, Japan); Sulev Haldre, MD (Tartu University Hospital, Tartu, Estonia); Khalid Hamandi, MD (University Hospital of Wales, Cardiff, UK); Lyubomir Haralanov, MD (SHATCVD, National Heart Hospital, Sofia, Bulgaria); Satoru Hiroshima, MD (Asahikawa Medical University Hospital, Asahikawa, Japan); Edouard Hirsch, MD (Centre Hospitalier Universitaire de Strasbourg, Strasbourg, France); Koji Iida, MD (Hiroshima University Hospital, Hiroshima, Japan); Jan Ilkowski, MD (NZOZ NEURO-KARD Ilkowski i Partnerzy Sp. Partnerska Lekarzy, Poznan,

Poland); Kousuke Kanemoto, MD (Aichi Medial University Hospital, Nagakute, Japan); Sasho Kastrev, MD (MHAT Puls, Blagoevgrad, Bulgaria); Mark Keezer, MD (Centre de Recherche du Centre Hospitalier de l'Université de Montréal, Montreal, QC, Canada); Anna Kelemen, MD (Országos Klinikai Idegtudományi Intézet, Budapest, Hungary); Christoph Kellinghaus, MD (Klinikum Osnabrück, Epilepsiezentrum Münster-Osnabrüeck, Osnabrück, Germany); Vasilios Kimiskidis, MD (Aristotle University of Thessaloniki, AHEPA University Hospital, Thessaloniki, Greece); Demet Kinay, MD (Türkiye Cumhuriyeti Sağlik Bakanliği Okmeydani Education and Research Hospital, Istanbul, Turkey); Masako Kinoshita, MD (Utano National Hospital, Kyoto, Japan); Pavel Klein, MD (Mid-Atlantic Epilepsy and Sleep Center, Bethesda, MD, USA); Agata Klosek, MD (Twoja Przychodnia, Centrum Medyczne Nowa Sol, Nowa Sol, Poland); Susanne Knake, MD (Universitätsklinikum Giessen und Marburg GmbH, Marburg, Germany); Jitka Kocvarova, MD (Fakultní nemocnice u sv. Anny v Brne, Brno, Czech Republic); Shinji Kondo, MD (Sanin Rosai Hospital, Tottori, Japan); Gregory Krauss, MD (The Johns Hopkins Hospital, Baltimore, MD, USA); Albrecht Kunze, MD (Universtitätsklinikum Jena, Jena, Germany); Kenneth Laxer, MD (California Pacific Medical Center, San Francisco, CA, USA); Martine Lemesle-Martin, MD (Centre Hospitalier Universitaire de Dijon, Dijon, France); Holger Lerche, MD (Universitätsklinikum Tübingen, Tübingen, Germany); Ande Lindmae, MD (North ESTONIA Medical Centre, Tallinn, Estonia); Alicja Lobinska, MD (NZOZ IGNIS Dr. Med. Alicja Lobinska, Swidnik, Poland); Elmer Lopez Meza, MD (Neurociencias Estudios Clínicos S.C., Culiacán, Mexico); Taketoshi Maehara, MD (Tokyo Medical and Dental University, Tokyo, Japan); Ruta Mameniskiene, MD (Vilnius University Hospital Santaros Klinikos, Vilnius, Lithuania); Ainhoa Marinas Alejo, MD (Hospital de Cruces, Barakaldo, Spain); Meritxell Martínez Ferri, MD (Hospital Universitari MútuaTerrassa, Barcelona, Spain); Petr Marusic, MD (Fakultní nemocnice v Motole, Prague, Czech

Republic); Dimitar Maslarov, MD (1st MHAT, Sofia, Bulgaria); Andreu Massot-Tarrús, MD (Hospital General Universitario Gregorio Marañón, Madrid, Spain); Sophie Mathais, MD (Centre Hospitalier Universitaire de Clermont-Ferrand, Hôpital Gabriel Montpied, Clermont-Ferrand, France); Oriano Mecarelli, MD (Università degli Studi di Roma La Sapienza, Roma, Italy); Andrea Menes, MD (Debreceni Egyetem Kenézy Gyula Egyetemi Kórház, Debrecen, Hungary); Nicola Biagio Mercuri, MD (Azienda Ospedaliera Universitaria Policlinico Tor Vergata, Università Roma Tor Vergata, Roma, Italy); Alfred Meurs, MD (UZ Gent, Gent, Belgium); Roberto Michelucci, MD (Università di Bologna, Ospedale Bellaria, Bologna, Italy); Seyed Mohammad Mirsattari, MD (London Health Sciences Center, London, ON, Canada); Gabriel Möddel, MD (Westfälische Wilhelms-Universität Münster, Münster, Germany); Hidetoshi Nakamoto, MD (TMG Asaka Medical Center, Asaka, Japan); Tomas Nezadal, MD (Institut neuropsychiatrické péče, Prague, Czech Republic); Hirotomo Ninomiya, MD (Itami City Hospital, Itami, Japan); Soheyl Noachtar, MD (Klinikum der Universität München, Campus Grosshadern, München, Germany); Terence O'Brien, MD (Alfred Health, Melbourne, VIC, Australia); Taku Ochiai, MD (Ochiai Brain Clinic, Saitama, Japan); Ilona Palka-Kisielowska, MD (Centrum Medyczne Pratia Katowice, Katowice, Poland); Hemant Pandey, MD (MD First Research Chandler, Chandler, AZ, USA); Terry Peery, MD (Central Texas Neurology Consultants, Round Rock, TX, USA); Jukka Peltola, MD (Tampere University Hospital, Tampere, Finland); Shankar Perumal, MD (Novant Health Presbyterian Medical Center, Charlotte, NC, USA); Plamen Petkov, MD (Medical Center Vita 1 Ltd, Pleven, Bulgaria); Anna Piatek, MD (Clinical Research Center Sp. z o.o., Medic-R Sp. K., Poznan, Poland); Robert Powell, MD (Morriston Hospital, Swansea, UK); Monica Puligheddu, MD (Azienda Ospedaliera Universitaria di Cagliari, Cagliari, Italy); Pier Paolo Quarato, MD (Istituto Neurologico Mediterraneo Neuromed è un Istituto di Ricovero e Cura a Carattere Scientifico (IRCSS), Pozzilli, Italy); David Reutens, MD (Royal Brisbane

and Women's Hospital, Herston, QLD, Australia); Rodrigo Rocamora Zúñiga, MD (Hospital del Mar, Barcelona, Spain); Joanne Rogin, MD (Minneapolis Clinic of Neurology Ltd, Golden Valley, MN, USA); Felix Rosenow, MD (Universitätsklinikum Frankfurt, Frankfurt am Main, Germany); Inder Sawhney, MD (Morriston Hospital, Swansea, UK); Andreas Schulze-Bonhage, MD (Universitätsklinikum Freiburg, Freiburg, Germany); Eric Segal, MD (Northeast Regional Epilepsy Group, Hackensack, NJ, USA); Udaya Seneviratne, MD (Monash Medical Centre, Monash Health, Clayton, VIC, Australia); Pedro Jesús Serrano Castro, MD (Hospital Regional Universitario de Málaga, Málaga, Spain); Jerry Shih, MD (University of California, San Diego, La Jolla, CA, USA); Hae Won Shin, MD (North Carolina Translational and Clinical Sciences [NC TraCS] Institute, Chapel Hill, NC, USA); Jaysingh Singh, MD (Ohio State University, Columbus, OH, USA); Dzevdet Smajlovic, MD (University Clinical Centre Tuzla, Tuzla, Bosnia and Herzegovina); Dragoslav Sokic, MD (Clinical Center of Serbia, Belgrade, Serbia); Michael R. Sperling, MD (Comprehensive Epilepsy Center at Thomas Jefferson University, Philadelphia, PA, USA); Bernhard Steinhoff, MD (Kork Epilepsy Centre, Kehl-Kork, Germany); Hans-Beatus Straub, MD (Epilepsie-Zentrum Berlin-Brandenburg, Berlin, Germany); Enra Suljic, MD (Clinical Center University of Sarajevo, Sarajevo, Bosnia and Herzegovina); Charles Szabo, MD (The University of Texas Health Science Center, San Antonio, TX, USA); Andrzej Szczudlik, MD (Krakowska Akademia Neurologii Sp. z o.o., Krakow, Poland); Rafael Toledano Delgado, MD (Hospital Universitario Ramón y Cajal, Madrid, Spain); Sanja Tomasović, MD (Klinička bolnica Sveti Duh, Zagreb, Croatia); Marzena Tomaszewska, MD (Mazowieckie Centrum Badań Klinicznych s.c., Grodzisk Mazowiecki, Poland); Annelies Van Dycke, MD (AZ Sint-Jan Brugge-Oostende, Campus Sint Jan, Bruges, Belgium); Vicente Villanueva Haba, MD (Hospital Universitari i Politecnic La Fe, Valencia, Spain); David Vossler, MD (UW Valley Medical Center, Renton, WA, USA); Pascal Vrielynck, MD (Centre Hospitalier

Neurologique William Lennox, Ottignies, Belgium); Florian Weissinger, MD (Vivantes Humboldt-Klinikum, Berlin, Germany); Manori Wijayath, MD (Westmead Hospital, Westmead, NSW, Australia); Seher Naz Yeni, MD (Istanbul University-Cerrahpaşa Medical Faculty, Istanbul, Turkey); Christian Zentner, MD (Prince of Wales Hospital, Randwick, NSW, Australia); Tomasz Zielinski, MD (NZOZ Novo Med, Katowice, Poland).
